# Theta power reduction and theta–gamma coupling desynchronization are associated with working memory interference and anxiety symptoms in panic disorder: a retrospective study

**DOI:** 10.1186/s12888-024-06272-3

**Published:** 2024-12-03

**Authors:** Ji Seon Ahn, Hye-Jin Hong, Jee Hang Lee, Jin Young Park

**Affiliations:** 1 Institute of Behavioral Science in Medicine, Yonsei University College of Medicine, Seoul, South Korea; 2 Department of Psychiatry, Yonsei University College of Medicine, Yongin Severance Hospital, Yonsei University Health System, 363 Dongbaekjukjeon-Daero, Giheung-Gu, Yongin, 16995 South Korea; 3 Center for Digital Health, Yongin Severance Hospital, Yonsei University Health System, Yongin, South Korea; 4 Department of AI & Informatics, Sangmyung University, Seoul, South Korea; 5 Department of Human-Centered AI, Sangmyung University, 20 Hongjimun 2-Gil, Jongno-Gu, Seoul, 03016 South Korea

**Keywords:** Panic disorder, Anxiety, Working memory, Mental arithmetic, Functional interaction, Theta–gamma coupling, Theta

## Abstract

**Background:**

Theta-gamma coupling (TGC) describes the modulation of gamma oscillations by the theta phasic activity, which is crucial for processes such as the ordering of information during working memory (WM) performance. The mental arithmetic (MA), which involves performing calculations with numbers, is a crucial tool for evaluating and understanding the sensory processing and management abilities of WM. Evaluating TGC may provide greater insight into the neural mechanisms mediating WM deficits in panic disorder (PD).

**Methods:**

Medical and electroencephalography (EEG) records of psychiatric outpatient clinic between 1 March 2020 and 30 September 2023 were retrospectively reviewed. A total of 34 PD patients and 34 age- and sex-matched healthy controls (HCs) underwent EEG to assess the overall functional interaction of the brain using multi-channel EEG analysis, focusing on specific brain regions including the frontal, temporal, parietal, and occipital lobes. EEG recordings were conducted during two sessions: a 5-min eyes-closed resting-state (RS) and a subsequent 5-min eyes-closed MA. The TGC and the spectral power of the theta and gamma frequency bands, which are well known to be associated with WM, were analysed.

**Results:**

Compared to those in HCs, TGC and theta power were significantly attenuated in PD patients. When analysing both HCs and PD patients together, RS TGC and relative theta power were negatively correlated with state anxiety and perceived stress scores, respectively. In contrast, TGC and relative theta power during the MA condition were positively correlated with the MA performance. Specifically, in PD patients, RS theta power across all electrodes was significantly negatively correlated with the Hamilton Anxiety Scale (HAMA) score. Linear regression analysis revealed that theta power in the T5 channel remained negatively correlated with pathological anxiety as measured by the HAMA score, even after controlling for other confounding factors.

**Conclusions:**

This study highlights significant alterations in TGC and theta power in PD patients. PD patients exhibit reduced TGC and theta power compared to HCs, indicating deficits in the neural mechanisms underlying anxiety and/or WM in PD. These insights contribute to a better understanding of the neural basis of WM deficits in PD and suggest potential avenues for targeted therapeutic interventions.

**Supplementary Information:**

The online version contains supplementary material available at 10.1186/s12888-024-06272-3.

## Background

Panic disorder (PD) is a common type of anxiety disorder (AD) that includes generalized anxiety disorder (GAD) and social anxiety disorder (SAD) [1]. All three conditions—PD, GAD, and SAD—are types of anxiety disorders; however, they exhibit distinct psychological and physiological mechanisms. PD is characterised by sudden, intense panic attacks due to amygdala and prefrontal cortex dysregulation; GAD involves chronic worry linked to hypothalamus–pituitary–adrenal axis activation and altered amygdala-prefrontal connectivity; SAD entails fear of social situations with heightened amygdala activity and disrupted prefrontal cortex function [2]. Despite their high lifetime prevalence, the pathophysiological mechanisms and disease-specific alterations of these disorders remain largely unclear [2–4]. Differentiating and understanding the neural and pathophysiological mechanisms underlying PD, GAD, and SAD is crucial for both clinical and theoretical advancements, as it can inform better treatment strategies and enhance our comprehension of the disorder’s underlying processes, ultimately leading to better patient care.

Working memory (WM) is a key cognitive function often associated with anxiety [5, 6]. It involves the ability to retain, manipulate, and update information that is no longer present in the external environment, supporting tasks such as retaining sequences of numbers and solving mathematical problems [7, 8]. This function is essential for many cognitive processes and for guiding behaviour towards achieving goals [9]. Although some studies have reported deficits in WM performance in patients with anxiety disorders, the findings are inconsistent [10–14]. Some research indicates that PD patients do not exhibit WM impairments [15, 16], while other studies suggest deficits in this cognitive ability [5, 10, 17, 18]. The importance of investigating WM in PD lies in its significant impact on daily life and quality of life. Defective WM implies a diminished capacity for accessing, maintaining, and/or retrieving information. WM impairments can hinder everyday tasks, from basic activities to complex problem-solving, leading to functional impairment [19], which includes limitations in an individual’s ability to perform social, physical, and interpersonal activities. This, in turn, can result in a diminished quality of life and impose serious social burdens on individuals with PD [20–23].

Evaluation of functional connectivity (FC) in the brain is pivotal for gaining insights into cognitive functions and the mechanisms underlying brain disorders [24–28]. Neurophysiological research indicates that quantitative electroencephalography (EEG) provides valuable insights into the neural correlates of sensory and cognitive information processing, as well as the correlation between fundamental cortical brain mechanisms and psychological states [29, 30], serving as a biomarker of brain function [31–33]. Unlike magnetic resonance imaging (MRI), EEG enables real-time observation and analysis of the brain’s functional interactions, offering a dynamic view of neural activity [34].

Studies of neuro-psychological capabilities have revealed that specific oscillatory rhythms and wave synchronization are closely associated with various cognitive processes, including WM [35]. Understanding the role of these oscillatory rhythms is crucial in the context of PD, as they can provide insights into the underlying neural mechanisms that contribute to the disorder’s symptoms. Brain wave synchronisation, considered a mechanism for increased communication between cerebral areas, is particularly relevant in this regard [36, 37]. In PD, disruptions in theta wave synchronisation have been linked to impairments in memory consolidation and long-term memory flexibility [38, 39]. The role of theta waves is especially important for understanding learning and memory processes involving the medial prefrontal cortex and the hippocampus, areas known to be affected in PD [40]. These disruptions can exacerbate the cognitive deficits experienced by individuals with PD, making it difficult for them to manage stress and anxiety effectively. Therefore, investigating these oscillatory rhythms and their impact on cognitive functions in PD can not only enhance our theoretical understanding of the disorder but also inform the development of targeted interventions aimed at improving cognitive and overall brain function in PD patients.

A characteristic of the brain’s functional interactions is neuronal synchronisation, encompassing the interdependencies and dynamic interactions among different neural assemblies [41]. Cross-frequency coupling (CFC) in EEG recordings enables assessment of FC that extends beyond single-frequency evaluations of oscillatory activity, offering insights into how local neural networks process information across frequency ranges [41–43]. Phase-amplitude coupling (PAC), a type of CFC, reflects the interactions between cognitive functions and inter-areal communication by showing how the phases of slower oscillations modulate the amplitude of faster oscillations [41, 44, 45]. Theta-phase gamma-amplitude coupling (TGC) is a well-known example of this interaction and is suggested to be a neurophysiological process underlying WM.

Uncovering the association between anxiety and the neural correlates of WM function has important implications for understanding how anxiety impairs cognition and for developing novel interventions targeting anxiety-related cognitive impairments. To date, no published studies have correlated PD with TGC, which could be crucial for understanding how anxiety interferes with brain area communication and impacts WM. WM is vital for coordinating arithmetic-specific skills during tasks, but few neuroimaging studies have explored how mental arithmetic (MA) impacts brain activation patterns associated with number processing and arithmetic task performance. In this study, we investigated whether PD patients exhibit WM impairment and whether the severity of anxiety symptoms (measured by Hamilton Rating Scale for Anxiety [HAMA]) affects performance on mental arithmetic tasks. We hypothesised that PD patients would show:

1. Pathological anxiety, indicated by higher HAMA scores compared to healthy controls (HCs),
2. Defective working memory, indicated by decreased performance on MA compared to HCs, and
3. A disturbance in inter-areal communication, indicated by lower TGC compared to HCs.

Our hypothesis is based on documented WM impairments in PD [46, 47], and the relationship between TGC and WM [48, 49]. Therefore, the aims of the present study are to analyse differences between PD patients and HCs in HAMA scores, MA scores, and TGC to determine if high anxiety severity (indicated by higher HAMA scores) affects WM in PD.

## Methods

### Study design

This retrospective study screened patients who visited a psychiatric outpatient clinic for anxiety between 1 March 2020 and 30 September 2023, based on the medical records and EEG data.

### Sampling method

The sample size was determined using G*Power software for an independent t-test comparing PD patients (*n* = 34) with age- and sex-matched healthy controls (HCs, also *n* = 34). The goal was to detect a medium effect size (Cohen’s d = 0.7) with 80% power at a significance level of 0.05 (two-tailed).

### Participants

Sixty-eight participants were included in the final analysis. The PD group consisted of 34 patients with a diagnosis of PD by experienced psychiatrists using the criteria of the Structured Clinical Interview for the Diagnostic and Statistical Manual of Mental Disorders, 5th edition [50]. The inclusion criteria were individuals aged 19 to 65 years who underwent EEG and clinical psychological tests at the Department of Psychiatry, Yongin Severance Hospital. Exclusion criteria included the presence of brain damage or neurological disorders, schizophrenia or mood disorders accompanied by psychosis, bipolar spectrum disorders, schizophrenia spectrum disorders, autism spectrum disorders, and serious medical conditions. The HC group consisted of 34 sex- and age-matched HCs, recruited through posters at the hospital and announcements on a website. Psychiatric diagnoses for the HCs were assessed using the Mini-International Neuropsychiatric Interview, which was administered by either a psychiatrist or trained graduate-level psychologists [51]. EEG recordings were performed at the Department of Psychiatry and the outpatient clinic. The inclusion criteria for HCs were individuals aged 19 to 65 years who underwent EEG recordings and clinical psychological tests at the Department of Psychiatry, Yongin Severance Hospital. Exclusion criteria for HCs included age younger than 19 years, the presence of brain lesions or a history of neurological or psychiatric disorders, use of psychiatric medications, and being deemed unable to participate in the study by a psychiatrist. All HCs were fully informed about the purpose and procedures of the study, and they provided written informed consent. The study was conducted in accordance with the Declaration of Helsinki and was reviewed and approved by the Institutional Review Board of Yongin Severance Hospital, Yonsei University (approval number: 9–2022-0199; approval date: 20 February, 2023).

### Measures

#### Clinical assessment

Clinical assessments included the HAMA and Hamilton Rating Scale for Depression (HAMD), the State-Trait Anxiety Inventory (STAI), and the Perceived Stress Scale (PSS). The HAMA assessed anxiety severity with 14 symptom-oriented questions rated from 0 (not present) to 4 (very severe). Scores 0–17 indicated mild anxiety, 18–25 mild to moderate anxiety, and 26–30 moderate to severe anxiety [52]. The HAMD is a multiple-choice questionnaire that clinicians use to rate the severity of a patient’s depression. A score of 0–7 is considered to be normal, and scores of 20 or more indicate moderately severe depression [53]. Cronbach’s alphas for the HAMA and HAMD subscales were 0.870 and 0.857, respectively [54]. The STAI assessed anxiety levels with 20 items each for state and trait anxiety. The core attributes primarily assessed by the STAI-S scale are tension, anxiety, and worry. Scores ranged from 20 to 80, with ≥ 52 indicating significant anxiety symptoms [55]. Cronbach’s α for the reliability of the STAI-S was 0.92 [56]. Trait anxiety was excluded to focus on current anxiety states. The PSS evaluated perceived stress levels using a10-item questionnaire on a 5-point Likert scale (0–4), with higher scores indicating greater stress (Cronbach’s α = 0.82) [57, 58].

#### Experimental procedures

Participants in the HC group were instructed to minimize the ingestion of stimulant medications (including but not limited to caffeine) and other substances known to affect brain activity as much as possible in the 48 h preceding the experimental session, following protocols established in our previous research [59]. While complete abstinence may be challenging, efforts were made to mitigate the potential effects of these substances on brain activity during EEG recordings. Each participant underwent EEG recordings while comfortably seated in an electrically shielded, sound-attenuated room. They were instructed to maintain a natural and comfortable resting posture throughout the procedure. Before commencing EEG recording, participants were instructed to close their eyes, remain still in the chair, relax, and stay awake. They were encouraged to relax their jaw muscles and minimise ocular and other movements. The experimental protocol consisted of two sessions, each lasting 5 min. The first session involved a resting state (RS) EEG recording with participants keeping their eyes closed. This was followed by a second session during which participants performed a MA with their eyes closed, serving as a WM task in this study. The MA was specifically designed with five difficulty levels, varying by the number of digits in the arithmetic problems presented. At the first level, participants were tasked with adding two one-digit numbers (e.g., 4 + 6). Subsequent levels increased in difficulty, with one number progressively containing more digits. For example, the highest level involved adding two three-digit numbers (e.g., 352 + 885). Participants were instructed to perform mental calculations for each digit that was auditorily presented and to achieve a high score by answering as many arithmetic problems as accurately as possible within the given time frame. The total score was calculated based on the number of correct responses provided at the highest level of difficulty. Performance in the MA was evaluated based on the average accuracy of responses across all difficulty levels. The average MA score (number of correct responses) was compared between the PD and HC groups.

#### EEG data acquisition

EEG data were recorded continuously using Net Station version 5.2 software (Electrical Geodesics, Eugene, OR) and a 64-channel HydroCel Geodesic Sensor Net (Electrical Geodesics).The electrode position follows the modified international 10–20 system, known as the 10–10 electrode distribution system (Applied Neuroscience, St. Petersburg, FL). Placement included 57 recording electrodes (AF4, F2, FCz, Fp2, Fz, FC1, AFz, F1, FP1, AF3, F3, F5, FC5, FC3, C1, F7, FT7, C3, CP1, C5, T3, TP7, CP5, P5, P3, A1, T5, P1, P9, PO3, Pz, O1, POz, Oz, PO4, O2, P2, CP2, P4, P10, T6, P6, CP6, A2, TP8, C6, C4, C2, T4, FC4, FC2, FT8, FC6, F8, F6, F4, Cz). All EEG channels were referenced to the scalp vertex electrode (Cz). Sponge-based carbon fiber electrodes (Ag/AgCl-coated and carbon-filled plastic electrodes with a sponge) were placed on the scalp in a high-density array. Before the sensor net was applied, the sponges were soaked in a solution of 5 mL/L of baby shampoo and 6 mL KCl/L of distilled water to facilitate electrical contact between the scalp and the electrodes. All electrode impedances were maintained at < 50 kΩ according to the guidelines of Electrical Geodesics. The EEG data were digitised and amplified at a sampling rate of 1 kHz using the Geodesic EEG system 400 (Electrical Geodesics.).

#### EEG data preprocessing

The EEG data were pre-processed and analysed offline using a custom script (MATLAB 2020a; MathWorks, Natick, MA) and the EEGLAB toolbox [60]. Wavelet-enhanced independent component analysis (ICA) and the multiple artifact rejection algorithm were employed for semi-automated preprocessing and artifact correction. The continuous EEG data were re-referenced to an average reference and filtered using a 0.5–200 Hz bandpass filter and notch filters at 60, 120, and 180 Hz. A total of 57 EEG channels were examined in our study. Channels with error probabilities exceeding three standard deviations from the mean were deemed “bad channels” and removed [61]. This evaluation was performed twice for each data file. Spherical interpolation from neighbouring channels with Legendre polynomials up to the seventh order of their signals was used to replace the omitted data channels. Spherical interpolation was chosen to ensure the continuity and spatial consistency of the EEG data, as this method effectively estimates the signal values of the bad channels by considering the signals from surrounding channels, thereby preserving the overall data integrity. Components with artifact probabilities > 0.8 were eliminated using ICA, an established method for effectively isolating ocular, electromyographic, and electrocardiographic artifacts [62]. The processed and cleaned data were saved in the EEGLAB file format (.set files).

### Power spectra analysis

The spectral power of the EEG data was calculated for each participant via fast Fourier transformation using the signal processing toolbox in MATLAB. Time windows of 4000 ms with an 8-ms overlap were used for spectral analysis. A Hamming window was applied to minimize spectral leakage. The following frequency bands were set for spectral analysis: theta (4–8 Hz) and gamma (30–50 Hz). These frequency bands were selected based on recent studies showing that theta and gamma oscillations play an important functional role in long-range cortical network activity and WM [63, 64]. The absolute powers of the theta and gamma bands were averaged over all the time windows and frequency bands for further analyses [41]. The relative power was calculated by dividing the absolute power in each frequency band by the percentage of absolute power summed over the frequency bands.

### TGC analysis

The strength of the CFC between the low-frequency theta phase and amplitude of gamma oscillations was assessed using the modulation index (MI) approach [65]. The MI quantifies the deviation of the phase–amplitude distribution from a uniform distribution (MI = 0). A higher MI value indicates stronger coupling, implying lower entropy H(P). To calculate the MI value, clean data from 20–200 s (corresponding to 5–50 epochs) were subjected to PAC analysis. For TGC computation, data were filtered into theta and gamma bands. This process separates the main periodic signals used in TGC calculations by emphasizing the frequency characteristics in each band. Standard Hilbert transform was applied to the time series representing the theta phase and gamma amplitude envelope. Subsequently, a composite time series was constructed, aligning the gamma oscillations with each phase of the theta rhythm. The theta phase was divided into 72 bins of 5º, ranging from ˗180º to 180º, and the mean amplitudes of the gamma oscillations were computed for each phase bin. These values were normalised by dividing the mean amplitude of each phase by the sum of all bins. The MI was calculated using the following formula: MI = log(N) ˗ H(P) / log(N), where N, log(N), and H(P) represent the number of phase bins, entropy of uniform distribution, and entropy of P, respectively [65, 66].

### Statistical analysis

The demographic, clinical, and electrophysiological variables were summarised using descriptive statistics such as the mean and standard deviation when they were normally distributed; otherwise, they were expressed as the median and interquartile range after testing for normality. The Kolmogorov–Smirnov test was performed to determine the normal distribution of the data [67]. Comparative analyses were conducted between PD patients and HCs. Continuous variables were assessed using the parametric test (independent Student’s t-tests) or the non-parametric test (Mann–Whitney U test) while dichotomous variables were evaluated using chi-squared tests. A significance level of *p* < 0.05 was applied, with adjustment for multiple comparisons using the false discovery rate (FDR) method according to the Benjamini–Hochberg method [68]. This adjustment was performed across all 57 electrodes utilized in the individual EEG data analysis to mitigate the risk of Type I errors. Pearson’s correlation coefficients were employed to analyse associations between EEG measures and baseline characteristics of the two study groups. Significant correlations identified in these analyses were further investigated. Linear regression models were employed to explore relationships between EEG measures and the HAMA score in PD patients. The models included the HAMD score, daily dose of benzodiazepines (BDZ), and PSS as covariates. These analyses aimed to elucidate how EEG findings correlate with anxiety severity in PD. All analyses were conducted using MATLAB statistical toolbox version R2022a (MathWorks) and SPSS version 25.0 (IBM, Armonk, NY).

## Results

### Demographic and clinical characteristics

**Fig. 1:**
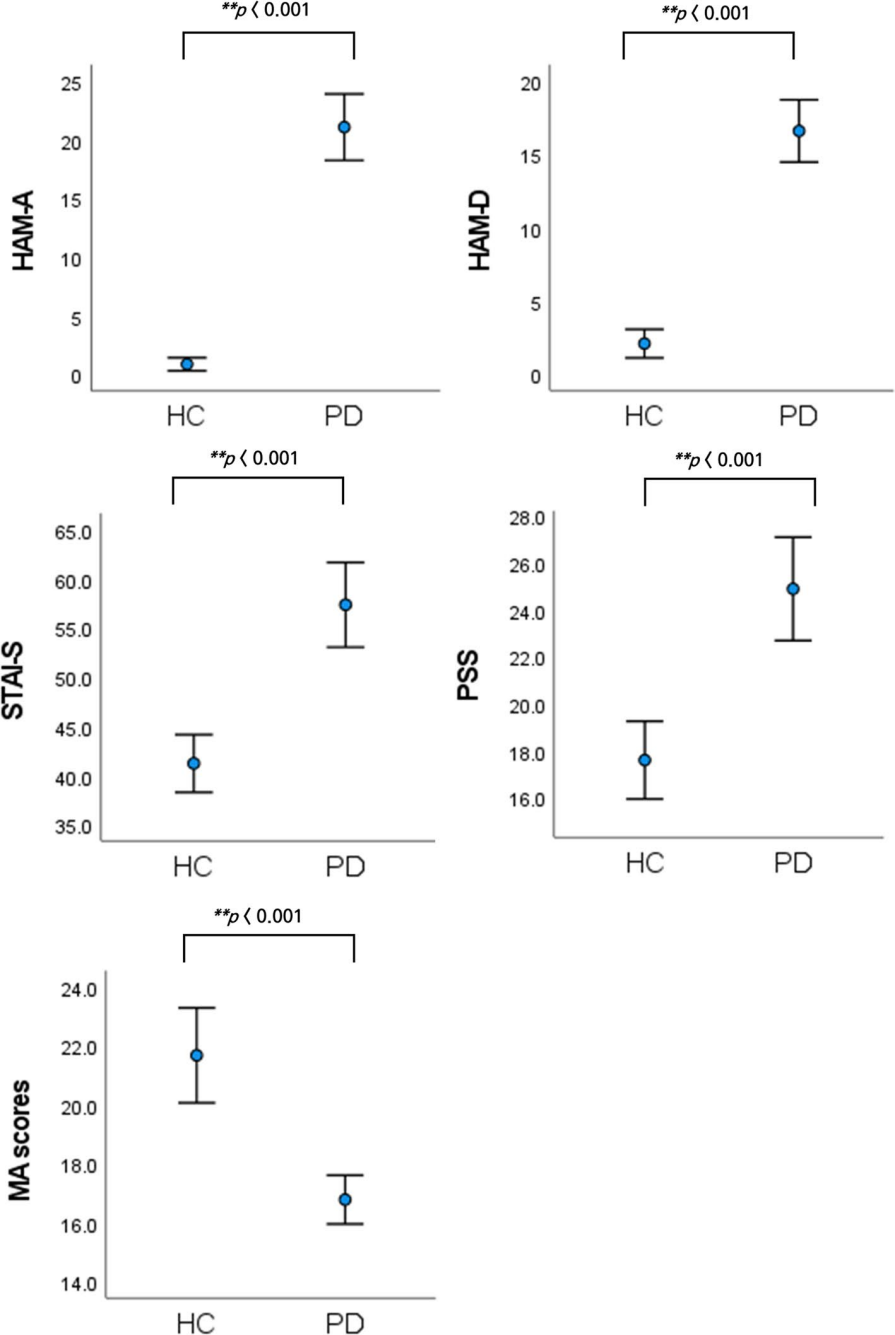
Comparison of mean scores between PD patients and HCs on HAMA, HAMD, STAI-S, and PSS (error bars represent 95% confidence intervals [CI]). PD, panic disorder; HC, healthy control; HAMA, Hamilton Rating Scale for Anxiety; HAMD, Hamilton Rating Scale for Depression; STAI, State-Trait Anxiety Inventory; PSS, Perceived Stress Scale

Thirty-four [14 (41.2%) male] PD patients, whose ages ranged from 20 to 41 years (mean age 26.21 ± 4.43 years), and 34 [19 (55.9%) male] HCs, whose ages ranged from 20 to 41 years (mean age 25.85 ± 4.30 years) were included in this study. Their clinical characteristics and MA results are described in Table 1. The PD patients and HCs were matched for age and sex, thus there were no significant between-group differences were observed with regards to age (*p* = 0.574) or sex (*p* = 0.332). The MA scores of PD patients significantly lower than that of HCs (*p* < 0.001), whereas the HAMA, HAMD, STAI-S, and PSS scores of PD patients were significantly higher than those of the HCs (all *p* < 0.001) (Table 1; Fig. 1). Most PD patients in this study were on pharmacotherapy such as antipsychotics or BDZs. We controlled for the potential alterations in EEG findings by these medications by standardising doses into equivalent amounts within specific drug categories.

**Table 1:** Demographic, clinical and medication data

Variables	PD (*n* = 34)	HC (*n* = 34)	Z/*χ*^*2*^	*p*
	Median (IQR) or *n* (%)			
Age, years	25.0 (23.75 ˗ 26.25)	25.0 (23.75 ˗ 28.25)	˗0.6	0.574
Male, n (%)	14 (41.2)	19 (55.9)	1.5	0.332
MA score	16.4(15.0 ˗ 18.0)	21.0 (19.0 ˗ 23.25)	˗ 5.5^*^	< 0.001
HAMA	20.0 (16.0 ˗ 26.25)	0.0 (0.0 ˗ 1.00)	˗7.2^*^	< 0.001
HAMD	16.0 (11.0 ˗ 21.25)	0.5 (0.0 ˗ 3.00)	˗7.1^*^	< 0.001
STAI-S	59.0 (48.5 ˗ 66.75)	42.1 (36.0 ˗ 47.25)	˗5.0^*^	< 0.001
PSS	24.9 (6.7)	17.6 (4.9)	˗4.9^*^	< 0.001
Drug use, n(%)				
No drug use	1 (2.9)			
Antidepressants	27 (79.4)			
BDZ	29 (85.3)			
Non-BDZ anxiolytics	2 (5.9)			
Antipsychotics	2 (5.9)			
Other	3 (8.8)			
Daily dose of BDZ^a^	1.99 (1.5)			

Differences between groups were tested using t-test for normally distributed data and expressed as mean and standard deviation; while the non-normal distributed data were analysed by Mann–Whitney U test and expressed as median and interquartile range. Antidepressants (escitalopram, fluoxetine, paroxetine, sertraline, duloxetine, venlafaxine, mirtazapine, bortioxetine, trazodone, sodium tianeptine); BDZs (alprazolam, clonazepam, etizolam, lorazepam, zolpidem); Non-benzodiazepine anxiolytics (buspirone); antipsychotics (aripiprazole, quetiapine); other (propranolol hydrochloride)

*Abbreviations*: *PD* panic disorder, *HC* healthy control, *MA* mental arithmetic, *HAMA* Hamilton Rating Scale for Anxiety, *HAMD* Hamilton Rating Scale for Depression, *STAI-S* state score of State-Trait Anxiety Inventory, *PSS* Perceived Stress Scale, *BDZ* benzodiazepine

^*^p < 0.001

^a^Data presented as oral lorazepam-equivalent dose for BDZs at the time of EEG recording. BDZ doses were calculated and transformed into lorazepam equivalents. Lorazepam 1 mg equivalent doses: diazepam 5 mg, clonazepam 0.375 mg, alprazolam 0.5 mg, etizolam 1 mg

### Comparison of relative power between PD patients and HCs

**Fig. 2:**
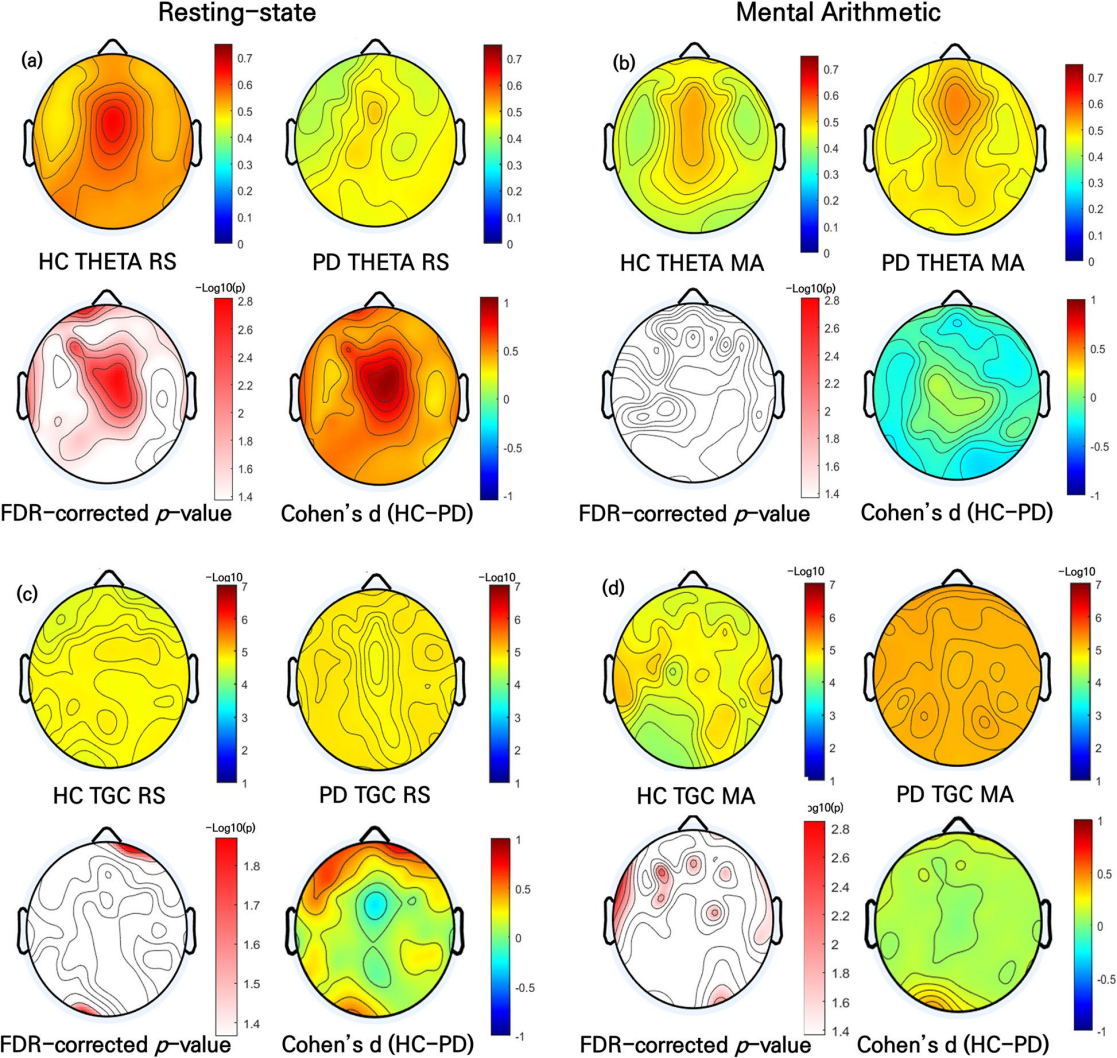
Topographic maps of relative theta power and TGC between PD patients and HCs. **a** Relative theta power in HCs (top left) and PD patients (top right) during the RS condition. **b** Relative theta power in HCs (top left) and PD patients (top right) during the MA condition. **c** TGC in HCs (top left) and PD patients (top right) during the RS condition. **d** TGC in HCs (top left) and PD patients (top right) during the MA condition. In each panel, topographical distribution of the corresponding FDR-corrected *p*-value obtained using a two-sample t-test (bottom left) and Cohen’s d (bottom right). TGC, theta–gamma coupling; PD, panic disorder; HCs, healthy controls; RS, resting-state, MA, mental arithmetic

After multiple comparisons correction, the PD patients demonstrated significantly decreased relative theta power in the frontal (AF4, F2, FCz, FP2, Fz, FC1, AFz, F1, FP1, AF3, F3, F5, FC5, FC3, F7, FT7, FC4, FC2, FT8, FC6, F8, F6 and F4), temporal (T3, TP7, T5 and T4), central (C1, C3, CP1, CP5, CP2, C4, C2 and Cz), and parietal (P5, P3, P1, P9, PO3, Pz, POz, PO4, P2 and P4) regions compared to the HCs during the RS condition (all *p* < 0.05 with FDR-corrected; Fig. 2a).

During the MA condition, compared with HCs, PD patients had a greater relative theta power at all electrodes except FC1, C1, CP1, CP2 and Cz; however, significant differences below the FDR threshold were not observed (Fig. 2b) (see Additional file 1—Supplementary Table 1).

### Comparison of TGC between PD patients and HCs

During the RS condition, PD patients exhibited significant attenuation of the MI of TGC compared with HCs, indicating altered connectivity. Significant differences were observed in the frontal (FP2) and occipital (O1) regions (all *p* < 0.05 with FDR-corrected; Fig. 2c).

During the MA condition, the MI of the TGC was significantly lower in PD patients than in HCs, with significant differences in the frontal (AF4, F2, FCz, FP2, Fz, AFz, F1, Fp1, AF3, F3, F5, FC5, FC3, F7, FT7, FC4, FT8, FC6, F8, F6, and F4), temporal (T3, T5, T6, TP8, and T4), central (C1, CP1, CP5, CP2, CP6, C6, C4, and C2), parietal (P5, P3, P1, P9, PO3, Pz, POz, PO4, and P6), and occipital (O1, Oz, and O2) regions(all *p* < 0.05 with FDR-corrected; Fig. 2d) (see Additional file 1—Supplementary Table 1).

### Correlation between EEG and clinical variables

**Fig. 3:**
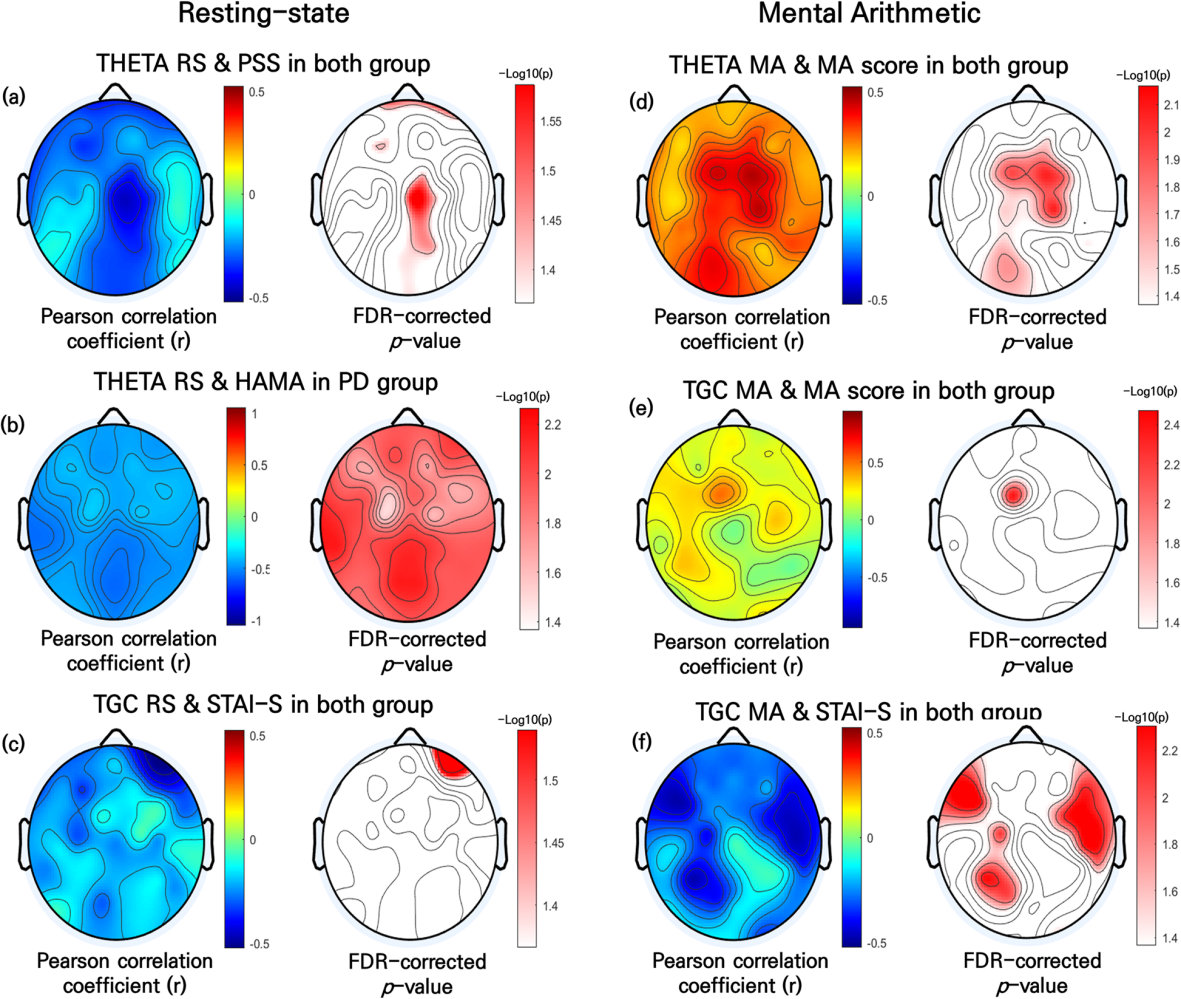
Topographical Representation of the Pearson’s Correlation Analysis Results for Relative Theta Power, TGC, Anxiety, and MA Scores in PD Patients and HCs. (**a**) Relative theta power during the RS condition and the PSS score in all subjects. (**b**) Relative theta power during the RS condition and the HAMA score in PD patients. (**c**) TGC during the RS condition and the STAI-S score in all subjects. (**d**) Relative theta power during the MA condition and MA scores in all subjects. (**e**) TGC during the MA condition and MA scores in all subjects. (**f**) TGC during the MA condition and the STAI-S score in all subjects. Left: Pearson’s correlation coefficients; right: false discovery rate-corrected p-values. TGC, theta–gamma coupling; PD, panic disorder; HC, healthy control; RS, resting-state; STAI, State-Trait Anxiety Inventory; PSS, Perceived Stress Scale; HAMA, Hamilton Rating Scale for Anxiety Additionally, there are some parts that were obscured in Figures 2 and 3, so I will submit the revised figures to ensure all elements are clearly visible

During the RS condition, Relative theta power was negatively correlated with the PSS scores. A significant correlation was found in the frontal (FP2, F1, FP1, F3, F7, and FC2), central (CP2, C2, and Cz), temporal (T3), and parietal (PO3, Pz, Poz, PO4, and P2) regions (all *p* < 0.05 FDR-corrected; Fig. 3a). In the PD group, relative theta power was significantly negatively correlated with the HAMA scores across all electrodes. (all *p* < 0.05 with FDR-corrected; Fig. 3b). TGC was negatively correlated with the STAI-S scores. A significant correlation was observed in the frontal (AF4 and FP2) regions (all *p* < 0.05 FDR-corrected; Fig. 3c).

During the MA condition, The relative theta power was positively correlated with MA scores. A significant correlation was observed in the frontal (F2, FCz, FC1, FC3, FC4, and FC2), central (C1, CP1, CP2, C4, C2, and Cz), temporal (T3), parietal (POz, and P6), and occipital (O1 and Oz) regions (all *p* < 0.05 with FDR-corrected; Fig. 3d). TGC was also positively correlated with MA scores. A significant correlation was identified in the frontal (FCz and FC1) region (all *p* < 0.05 with FDR-corrected; Fig. 3e). TGC was negatively correlated with the STAI-S scores as well. A significant correlation was observed in the frontal (AFz, F1, F5, FC5, FC3, F7, FT7, FC4, FC2, FT8, FC6, F8, and F6), central (C1, C3, CP1, C6, and C4), temporal (TP8), parietal (P3, P1, and PO3), and occipital (O2) regions (all *p* < 0.05 with FDR-corrected; Fig. 3f) (see Additional file 1—Supplementary Table 2).

### Association among EEG data, MA and clinical scale scores in PD patients

**Fig. 4:**
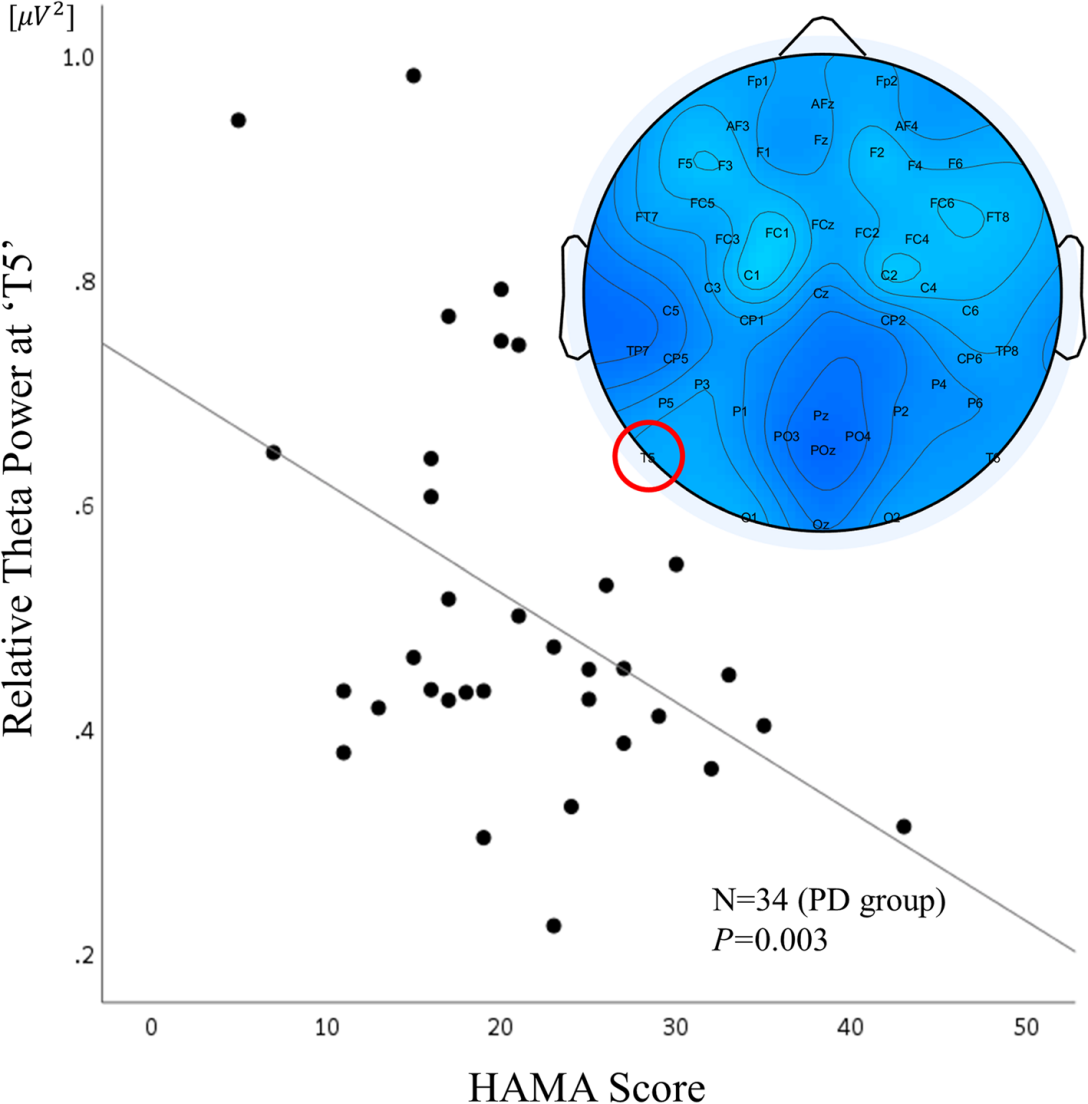
Association of left temporal relative theta power during the RS condition with the HAMA score in PD patients. Topographical representation of Pearson’s correlation coefficients. Correlation (linear fit) between relative theta power in the RS condition at T5 and the HAMA scores in PD patients (*n* = 34, *R*^*2*^ linear = 0.737, *p* = 0.003). RS, resting-state; HAMA, Hamilton Rating Scale for Anxiety; PD, panic disorder

Linear regression analysis was conducted to determine whether EEG data was independently associated with the HAMA score in PD patients after controlling for HAMD score, age, sex, and HAMD score. A significant regression equation was found at T5 [F(5,33) = 15.869; *p* < 0.001], yielding an *R*^*2*^ value of 0.737. The HAMA score exhibited an independent negative association with relative theta power during the RS condition at electrode T5 (*p* = 0.003; Fig. 4) and an independent positive association with the HAMD score (*p* < 0.001). By contrast, no significant associations were found with the daily dose of BDZ (*p* = 0.866), PSS score (*p* = 0.851). The RS relative theta power at T5 (ß = ˗14.885; *p* = 0.003) and HAMD score (ß = 0.968; *p* < 0.001) were significant predictors of the HAMA score in the model independently (Table 2).

**Table 2:** Linear regression predicting anxiety symptoms in PD patients

	ß	B (SE)	*t*	*p*
Model				< 0.001
(Constant)	11.98	4.39	2.72	0.011
HAMD	0.97	0.16	5.93^**^	< 0.001
Relative theta power (‘T5’)	˗14.89	4.57	˗3.26^*^	0.003
Daily dose of BDZ^a^	0.73	0.43	0.17	0.866
PSS	-0.03	0.18	-0.19	0.851

Linear regression analyses were performed with the HAMA score as the dependent variable and PSS, benzodiazepine equivalent dosage, HAMD score, and relative theta power as independent variables. Standardized regression coefficients (β), Unstandardized coefficients (B) and Standard Errors (SE)

*PD* Panic disorder, *HAMD* Hamilton Rating Scale for Depression, *BDZ* Benzodiazepine, PSS, Perceived Stress Scale; HAMA, Hamilton Rating Scale for Anxiety

^*^p < 0.05

^**^p < 0.001

^a^Data presented as oral lorazepam-equivalent dose for benzodiazepines. Benzodiazepine doses were calculated and transformed into lorazepam equivalents. Lorazepam 1 mg equivalent doses: diazepam 5 mg, clonazepam 0.375 mg, alprazolam 0.5 mg, etizolam 1 mg

## Discussion

To the best of our knowledge, this study represents the first exploration of functional interaction patterns using EEG during both RS and MA conditions in PD patients, comparing these patterns with those observed in HCs. Our investigation comprehensively assessed power spectra and TGC in these contexts, facilitating direct comparisons within and between the PD patients and HCs. Theta and gamma activity were specifically targeted because of their critical roles in WM processes.

Our study reveals several key findings. First, PD patients exhibited significantly heightened clinical anxiety symptoms as measured by HAMA scores and substantial deficits in WM performance, specifically in MA tasks, compared to HCs. In our study, PD patients scored notably lower on MA tasks compared to controls, indicative of impaired WM function. Second, relative to HCs, PD patients demonstrated diminished theta power and TGC across scalp regions. The results demonstrate significant differences in TGC between the PD patients and HCs during the RS and MA conditions, as well as significant differences in relative theta power between the PD patients and HCs during the RS condition. Third, we observed a negative correlation between stress- or anxiety-related outcomes and both relative theta power and TGC, while MA performance exhibited a positive correlation with these EEG measures. Lastly, temporal theta power showed a robust negative association with HAMA scores, even after adjusting for demographic and clinical covariates such as age, sex, medication use, PSS, and depression severity (HAMD score). These results underscore the intricate relationship between neurophysiological alterations and clinical manifestations in PD, suggesting that disruptions in theta and gamma activities may underlie both cognitive impairments and anxiety symptoms observed in these patients.

The results demonstrate significant differences in TGC between the PD and HC groups during both RS and MA conditions. Specifically, PD patients exhibited significantly lower TGC than HCs at the regional level, namely in the frontal and occipital regions, during the RS condition. During the MA, PD patients showed lower TGC than HCs at the global level, with significant differences observed across a wide range of regions, including the frontal, temporal, central, parietal, and occipital areas. These differences suggest a potential loss of long-range communication among brain regions in PD patients. Our findings are consistent with an functional MRI study that documented reduced small-worldness in PD patients, indicating a tendency towards decreased global and local network efficiency in PD [69, 70]. Furthermore, our results contribute to mounting evidence suggesting a connection between PD and disturbances in functional integration within local networks. These abnormalities appear to be associated with diminished information-carrying functions across critical brain regions in PD [69].

EEG studies have shown that cognitive tasks enhance global coherence among distant brain regions, indicating that oscillating neural activities create widespread neuronal assemblies essential for cognitive functions [71]. Theta-band oscillations transmit requirements and facilitate relevant coordination among different brain regions [63, 64]. A previous study reported that the synchronisation of the theta band can coordinate neural communication between multi-brain regions and contribute to the maintenance of short-term memories [41]. In the event of a high demand for cognitive and inhibitory control, frontal theta rhythms systematically modulate the amplitudes of occipital gamma waves, which synchronously align with the frontal theta rhythms to facilitate communication [72, 73], indicating that the long-range coherent network executing cognitive functions is coordinated in the time window of theta oscillations. Distant interactions facilitated by TGC across cortical regions, such as between the prefrontal cortex (PFC) and parieto-occipital areas, may be hindered by stress, cognitive load, or heightened sensory input. This hindrance could manifest as decoupled PAC and diminished performance [74]. This communication mechanism may be disrupted when inhibitory control declines in PD patients, as evidenced by the divergence of theta and gamma rhythms in PAC. Our findings are consistent with those of previous studies indicating reduced FC [25–27, 75] and altered synchronous activity in EEG patterns among PD patients [76–78]. Existing evidence has highlighted a robust association between WM and cross-frequency synchronisation, particularly the interplay between theta and gamma oscillations [41, 79, 80]. The observed TGC and theta power outcomes in our study exhibited a strong correlation with MA performance under WM task conditions.

WM is closely related to mathematical abilities regardless of age; higher mathematical proficiency correlates with better performance on working memory tasks. MA is valuable for probing essential cognitive processes, including information retrieval, control process execution, and information updating. This task is often regarded as a primary measure for assessing WM because its problem-solving nature engages WM components [81, 82]. DeStefano and LeFevre urged researchers to use MA as a primary task in WM studies, as solving problems involving multiple digits likely requires the interaction of all components of the WM system [81]. Numerous studies have reported augmentation of theta oscillations during intricate cognitive processes, including WM tasks such as MA [83, 84], episodic encoding and retrieval [85–87], and error processing [88, 89]. Nevertheless, our results contradict the expectation of an increase in theta activity during MA, as we observed a notable decline in theta spectral power during the MA compared to the RS (neutral control) condition.

Specifically, in HCs, theta activity decreased during the execution of the MA, and theta activity was significantly negatively correlated with PSS scores. Our findings of reduced relative theta power during the MA condition, contrary to the expected increase hypothesised in some studies [63, 88–91], raise intriguing questions about the underlying mechanisms. Several potential explanations merit consideration. Firstly, methodological differences across studies, such as variations in task complexity, duration, and participant characteristics, can influence theta activity outcomes. Specifically, our use of the MA as a WM task, known for its stress-inducing properties, aligns with prior research demonstrating theta reductions under stress conditions [91]. This suggests that the cognitive demands and stress elicited by the MA task may have modulated theta oscillations differently compared to less stressful WM paradigms. Moreover, individual differences in anxiety levels, as indicated by the PSS, could have contributed to the observed relative theta power decrease during the MA condition. Elevated anxiety has been associated with altered neural responses, including theta suppression, particularly in tasks requiring high cognitive effort and attentional control [92, 93]. Therefore, the stressor nature might have influenced theta dynamics, reflecting a trade-off between cognitive engagement and stress-induced modulation of neural oscillations.

In contrast to the observed decrease in theta activity during MA condition in HCs, our study found that in the PD patients, theta activity during MA condition was higher compared to HCs. However, frontal midline theta activity, which is associated with WM processes, was significantly lower in the PD patients. These findings indicate that frontal midline theta is not only an index of successful WM manipulation but also reflects efficient PFC functioning during WM [71, 91]. The discrepancy between overall theta activity and frontal midline theta suggests that while general theta activity may increase, the specific frontal midline theta activity crucial for WM may be compromised in PD patients, highlighting potential disruptions in PFC functions related to WM. Our results confirmed and extended previous neuroimaging studies [83, 84, 91] by showing that frontal midline theta is generated in the medial prefrontal cortex during mental calculation.

Our results also demonstrated theta attenuation in individuals with higher PSS or HAMA scores, which may be associated with sensory and cognitive information processing disruptions. Those more vulnerable to stress may experience reduced theta activity, potentially indicating difficulties in suppressing irrelevant information unrelated to attention and cognitive engagement. Furthermore, the observed reduction in theta power during MA underscores the task’s effectiveness in probing cognitive processes under stress conditions, revealing potential implications for understanding cognitive deficits. Future studies should systematically explore these dynamics across diverse populations and employ multimodal approaches to disentangle the complex interactions between stress, cognitive function, and neural oscillations.

This study has several notable limitations that warrant careful consideration. Firstly, its retrospective design, based on a review of medical records, restricts the generalisability of our findings and precludes the establishment of causal relationships between EEG activity patterns and clinical outcomes in PD patients. Retrospective designs are inherently susceptible to biases related to data collection and retrospective analyses, potentially impacting the robustness and reliability of our conclusions. Furthermore, our study assessed anxiety levels at a single timepoint, which limits our understanding of the dynamic fluctuations in anxiety symptoms over time and their relationship with EEG measures. Future prospective studies employing longitudinal designs could capture these temporal variations comprehensively, offering deeper insights into the interplay between anxiety severity and neural activity dynamics. This approach may also facilitate the development of personalised treatment strategies tailored to individual anxiety profiles. Secondly, we were unable to find a daily BDZ dose-dependent correlation in power spectra, TGC, and anxiety symptom severity within the current PD sample (see Additional file 1—Supplementary Table 5, 6), indicating that BDZs do not influence these relationships in our results. However, despite our efforts to standardise drug dosages and categories (see Additional file 1—Supplementary Table 4), the diverse range of medications and their interactions could not be fully accounted for in our analyses. It is important to note that various drug effects, including those of antidepressants, other medications, and potential drug interactions, could not be completely ruled out. The potential effects of pharmacotherapy on EEG measures in PD patients remain a significant concern. Future research should consider comparing drug-naïve PD patients or those with limited drug usage against HCs to better understand the specific effects of medications on EEG markers of neural activity and FC. Moreover, our study focused exclusively on adults aged between 20 and 41 years, excluding children, adolescents, and older adults. Given the potential differences in neural development and aging-related changes in brain function, the applicability of our findings to broader age groups may be limited. Including diverse age cohorts in future studies would help elucidate age-related variations in EEG biomarkers and their implications for understanding PD pathology across the lifespan.

Addressing these limitations will enhance the validity and generalisability of findings, providing a more nuanced understanding of the complex interplay between anxiety, neural dynamics, and cognitive function in PD. Future research should include children and adolescents to enable a more comprehensive understanding of these relationships. We have additionally examined the impact of depressive conditions on TGC. Notably, the results revealed no significant differences in TGC between individuals with PD exhibiting mild depression (HAMD score 0–17) and those with moderate depression (HAMD score 18–30) [53] (see Additional file 1—Supplementary Table 7). This suggests that, at least within the scope of our study, depressive conditions did not appear to influence the observed relationship. However, the sample size of our study is relatively small, making it hard to assess the potential confounding factors on this occasion. Thus, further investigation is needed to validate our finding. Lastly, our findings pertain to PD and cannot be extrapolated to other subtypes of anxiety disorders. Despite these limitations, our study validates and broadens previous indications that fronto-temporal theta activity serves as a valuable biomarker for exploring the interplay between anxiety and cognition in PD patients. Moreover, our findings suggest that TGC could serve as a viable EEG marker for assessing WM performance and identifying abnormal FC interactions. Notably, individuals who exhibited higher top–down PFC control (higher TGC) or better prefrontal brain function (higher theta) demonstrated enhanced inhibitory control in response to stress.

Our results support two main hypotheses. First, our results align with the notion that PD patients may struggle to effectively inhibit irrelevant distractions during RS conditions and various emotional and cognitive tasks. This impairment is particularly pronounced under conditions of heightened anxiety in stressful situations. Second, our findings lend further credence to the concept that stressors exacerbate dysfunction in interactions within and between large brain networks, increased mental workload, and heightened sensory demands in PD patients. This study presents the state of the art to highlight distinct aspects of symptomatology and cognitive function in PD patients through analyses of power spectra and TGC, respectively. Our research sheds light on PD-related effects on regional neuronal activation and global neuronal connectivity during both RS and MA conditions, providing evidence of alterations in FC strength in PD patients. We observed correlations between changes in WM ability in PD patients and alterations in the degree of interactions between active neuron clusters or FC, such as TGC. We propose that the MI of TGC serves as a valuable tool for evaluating the degree of interactions between active neuron clusters within brain networks.

### Clinical implications and potential applications

Our study illuminates the neurophysiological underpinnings of PD, offering insights into its clinical implications and potential therapeutic applications. We observed reduced theta power and TGC in PD patients, correlating with heightened anxiety symptoms and cognitive deficits. These findings suggest that EEG-based biomarkers could serve as objective measures of symptom severity and treatment response. They hold promise for tailoring personalised treatment strategies targeting specific neural circuits implicated in PD. Additionally, integrating neurophysiological data into clinical practice may enhance diagnostic accuracy and treatment monitoring. EEG measures, particularly theta power, could potentially serve as prognostic indicators of disease progression and treatment efficacy, supporting the development of novel therapeutic interventions. Further research is needed to validate these biomarkers across diverse patient populations and treatment settings. Longitudinal studies examining changes in EEG measures alongside clinical outcomes are crucial for advancing our understanding of PD pathophysiology and optimising treatment approaches.

## Conclusions

Our study underscores the neurophysiological and clinical significance of FC in PD. By demonstrating the utility of TGC and its relationship with WM performance, we deepen our understanding of the intricate interplay between neural network dynamics and cognitive functions in PD. These insights provide a foundation for developing targeted interventions aimed at improving cognitive function in PD patients. Methodologically, power spectra and TGC analyses coupled with EEG equipment may serve as digital biomarkers for the quick and accurate diagnosis of PD in clinical settings.

In conclusion, our study advances both theoretical knowledge and clinical practice in PD research by elucidating the neurobiological mechanisms underlying the disorder. This contributes to the development of targeted interventions that could enhance patient outcomes and quality of life.

## Supplementary Information

Supplementary Material 1.

Supplementary Material 2.

## Data Availability

Data is provided within the manuscript or supplementary information files.

## Abbreviations

PD: Panic disorder
AD: Anxiety disorder
GAD: Generalised anxiety disorder
SAD: Social anxiety disorder
WM: Working memory
FC: Functional connectivity
EEG: Electroencephalography
MRI: Magnetic resonance imaging
CFC: Cross-frequency coupling
PAC: Phase–amplitude coupling
TGC: Theta-phase gamma-amplitude coupling
MA: Mental arithmetic
HAMA: Hamilton Rating Scale for Anxiety
HC: Healthy control
HAMD: Hamilton Rating Scale for Depression
STAI-S: State anxiety scores of State-Trait Anxiety Inventory
PSS: Perceived Stress Scale
RS: Resting state
ICA: Independent component analysis
MI: Modulation index
FDR: False discovery rate
BDZ: Benzodiazepine
PFC: Prefrontal cortex
