# Supplementary material for: Theta power reduction and theta–gamma coupling desynchronization are associated with working memory interference and anxiety symptoms in panic disorder: a retrospective study
Source: BMC Psychiatry. 2024 Dec 3;24:875. doi: 10.1186/s12888-024-06272-3 (PMC11613674; doi:10.1186/s12888-024-06272-3)
Supplement: Supplementary file 1 — Supplementary Material 1. [file 12888_2024_6272_MOESM1_ESM.docx]

Supplementary Materials for

## Theta power reduction and theta–gamma coupling desynchronization are associated with working memory interference and anxiety symptoms in panic disorder: A retrospective study

Ji Seon Ahn^†^, Hye-Jin Hong^†^, Jee Hang Lee* and Jin Young Park*

^*^Corresponding author. E-mail: empathy@yuhs.ac, jeehang@smu.ac.kr

## List of contents

Supplementary Tables

Table S1. The comparisons of TGC and relative theta power between HCs and PD patients

Table S2. Pearson’s correlation coefficients between the EEG measures and clinical characteristics across PD patients and HCs

Table S3. Pearson’s correlation coefficients between the relative theta power and HAMA scores in PD patients

Table S4. General Demographic and Medication Details for PD patients

Table S5. Pearson's correlation coefficient for clinical and medication data (Spearman rho)

Table S6. Pearson’s correlation coefficients between EEG measures and daily dose of BDZ in PD patients

Table S7. The comparisons of TGC between PD patients with mild and moderate depression

**Supplementary Table 1.** The comparisons of TGC and relative theta power between HCs and PD patients

| Lead | | PD patients | | HCs | | t-value  (HC-PD) | FDR-corrected  *p-*value |
| --- | --- | --- | --- | --- | --- | --- | --- |
|  |  | Mean | SD | Mean | SD |  |  |
| Resting-state TGC  (MI) | AF4 | 1.339.E-05 | 6.131.E-06 | 2.111.E-05 | 1.659.E-05 | 2.596 | 0.096 |
|  | F2 | 1.345.E-05 | 5.574.E-06 | 1.803.E-05 | 6.381.E-06 | 1.513 | 0.227 |
|  | FCz | 2.147.E-05 | 2.339.E-05 | 1.412.E-05 | 2.492.E-05 | -1.638 | 0.190 |
|  | Fp2 | 1.445.E-05 | 1.146.E-05 | 3.133.E-05 | 1.923.E-05 | 3.537* | 0.017 |
|  | Fz | 1.794.E-05 | 2.061.E-05 | 2.195.E-05 | 7.846.E-06 | 0.794 | 0.558 |
|  | FC1 | 1.679.E-05 | 1.112.E-05 | 1.575.E-05 | 2.030.E-05 | -0.422 | 0.745 |
|  | AFz | 1.403.E-05 | 7.608.E-06 | 2.041.E-05 | 1.685.E-05 | 1.701 | 0.173 |
|  | F1 | 1.409.E-05 | 5.858.E-06 | 2.138.E-05 | 3.178.E-05 | 2.364 | 0.110 |
|  | Fp1 | 1.352.E-05 | 1.058.E-05 | 2.668.E-05 | 2.500.E-05 | 2.274 | 0.110 |
|  | AF3 | 1.387.E-05 | 6.355.E-06 | 2.470.E-05 | 2.142.E-05 | 2.439 | 0.110 |
|  | F3 | 1.612.E-05 | 1.559.E-05 | 2.449.E-05 | 3.055.E-05 | 1.791 | 0.159 |
|  | F5 | 1.368.E-05 | 6.620.E-06 | 2.817.E-05 | 1.504.E-05 | 2.696 | 0.096 |
|  | FC5 | 1.393.E-05 | 5.372.E-06 | 1.905.E-05 | 1.634.E-05 | 1.853 | 0.151 |
|  | FC3 | 1.292.E-05 | 4.412.E-06 | 1.692.E-05 | 1.109.E-05 | 1.372 | 0.285 |
|  | C1 | 1.445.E-05 | 5.940.E-06 | 1.675.E-05 | 2.135.E-05 | 1.048 | 0.415 |
|  | F7 | 1.558.E-05 | 1.570.E-05 | 2.371.E-05 | 2.621.E-05 | 1.737 | 0.172 |
|  | FT7 | 1.274.E-05 | 5.031.E-06 | 2.469.E-05 | 1.420.E-05 | 2.606 | 0.096 |
|  | C3 | 1.378.E-05 | 4.821.E-06 | 1.779.E-05 | 1.154.E-05 | 1.547 | 0.219 |
|  | CP1 | 1.421.E-05 | 5.497.E-06 | 1.701.E-05 | 1.630.E-05 | 1.256 | 0.320 |
|  | C5 | 1.272.E-05 | 5.595.E-06 | 1.658.E-05 | 8.397.E-06 | 1.296 | 0.311 |
|  | T3 | 1.643.E-05 | 1.696.E-05 | 1.541.E-05 | 1.780.E-05 | -0.296 | 0.811 |
|  | TP7 | 1.281.E-05 | 5.355.E-06 | 1.959.E-05 | 1.083.E-05 | 2.113 | 0.116 |
|  | CP5 | 1.320.E-05 | 5.166.E-06 | 1.782.E-05 | 1.246.E-05 | 2.211 | 0.110 |
|  | P5 | 1.315.E-05 | 6.247.E-06 | 1.910.E-05 | 7.786.E-06 | 2.448 | 0.110 |
|  | P3 | 1.381.E-05 | 4.585.E-06 | 1.532.E-05 | 3.205.E-05 | 0.956 | 0.465 |
|  | T5 | 1.320.E-05 | 5.062.E-06 | 1.742.E-05 | 1.035.E-05 | 2.109 | 0.116 |
|  | P1 | 1.415.E-05 | 5.887.E-06 | 1.793.E-05 | 3.426.E-05 | 1.815 | 0.157 |
|  | P9 | 1.318.E-05 | 5.167.E-06 | 2.440.E-05 | 1.243.E-05 | 1.887 | 0.151 |
|  | PO3 | 1.406.E-05 | 1.194.E-05 | 1.680.E-05 | 8.944.E-06 | 0.890 | 0.500 |
|  | Pz | 1.594.E-05 | 1.757.E-05 | 1.449.E-05 | 1.882.E-05 | -0.403 | 0.745 |
|  | O1 | 1.285.E-05 | 6.115.E-06 | 2.581.E-05 | 1.005.E-05 | 3.793* | 0.017 |
|  | POz | 1.496.E-05 | 1.386.E-05 | 1.619.E-05 | 1.648.E-05 | 0.398 | 0.745 |
|  | Oz | 1.307.E-05 | 5.037.E-06 | 2.193.E-05 | 8.510.E-06 | 2.979 | 0.059 |
|  | PO4 | 1.729.E-05 | 2.489.E-05 | 1.724.E-05 | 1.172.E-05 | -0.011 | 0.992 |
|  | O2 | 1.498.E-05 | 1.950.E-05 | 1.759.E-05 | 1.957.E-05 | 0.628 | 0.648 |
|  | P2 | 1.529.E-05 | 2.120.E-05 | 1.850.E-05 | 1.161.E-05 | 0.622 | 0.648 |
|  | CP2 | 1.538.E-05 | 1.276.E-05 | 1.923.E-05 | 1.545.E-05 | 1.242 | 0.320 |
|  | P4 | 1.296.E-05 | 5.251.E-06 | 1.921.E-05 | 1.125.E-05 | 2.218 | 0.110 |
|  | P10 | 1.238.E-05 | 5.090.E-06 | 1.638.E-05 | 1.117.E-05 | 1.868 | 0.151 |
|  | T6 | 1.239.E-05 | 4.476.E-06 | 1.598.E-05 | 1.131.E-05 | 1.719 | 0.172 |
|  | P6 | 1.322.E-05 | 6.198.E-06 | 1.757.E-05 | 1.617.E-05 | 1.932 | 0.144 |
|  | CP6 | 1.386.E-05 | 5.771.E-06 | 2.050.E-05 | 8.543.E-06 | 2.235 | 0.110 |
|  | TP8 | 1.408.E-05 | 5.751.E-06 | 2.047.E-05 | 1.166.E-05 | 1.970 | 0.138 |
|  | C6 | 1.522.E-05 | 7.982.E-06 | 1.669.E-05 | 1.209.E-05 | 0.590 | 0.648 |
|  | C4 | 1.436.E-05 | 6.331.E-06 | 1.744.E-05 | 9.908.E-06 | 1.291 | 0.311 |
|  | C2 | 1.490.E-05 | 6.531.E-06 | 1.736.E-05 | 9.242.E-06 | 1.183 | 0.344 |
|  | T4 | 1.768.E-05 | 2.120.E-05 | 1.514.E-05 | 8.372.E-06 | -0.597 | 0.648 |
|  | FC4 | 1.428.E-05 | 5.960.E-06 | 1.532.E-05 | 9.104.E-06 | 0.573 | 0.648 |
|  | FC2 | 1.686.E-05 | 1.491.E-05 | 1.484.E-05 | 1.568.E-05 | -0.635 | 0.648 |
|  | FT8 | 1.217.E-05 | 4.783.E-06 | 1.843.E-05 | 1.072.E-05 | 2.213 | 0.110 |
|  | FC6 | 1.359.E-05 | 6.081.E-06 | 1.417.E-05 | 1.563.E-05 | 0.267 | 0.819 |
|  | F8 | 1.510.E-05 | 1.440.E-05 | 2.266.E-05 | 1.623.E-05 | 1.998 | 0.136 |
|  | F6 | 1.406.E-05 | 6.077.E-06 | 2.085.E-05 | 1.478.E-05 | 2.263 | 0.110 |
|  | F4 | 1.400.E-05 | 1.135.E-05 | 2.088.E-05 | 1.153.E-05 | 2.089 | 0.116 |
|  | Cz | 2.034.E-05 | 2.817.E-05 | 1.943.E-05 | 1.583.E-01 | -0.162 | 0.887 |

| MA test  TGC (MI) | AF4 | 1.15.E-05 | 4.99.E-06 | 2.37.E-05 | 1.44.E-05 | 4.443^†^ | <0.001 |
| --- | --- | --- | --- | --- | --- | --- | --- |
|  | F2 | 1.11.E-05 | 4.15.E-06 | 2.17.E-05 | 1.68.E-05 | 3.405* | 0.003 |
|  | FCz | 1.35.E-05 | 4.86.E-06 | 1.85.E-05 | 9.43.E-06 | 2.580* | 0.018 |
|  | Fp2 | 1.03.E-05 | 4.13.E-06 | 3.04.E-05 | 2.39.E-05 | 4.594^†^ | <0.001 |
|  | Fz | 1.14.E-05 | 4.59.E-06 | 2.69.E-05 | 2.40.E-05 | 3.551* | 0.002 |
|  | FC1 | 1.27.E-05 | 5.90.E-06 | 1.89.E-05 | 1.75.E-05 | 1.860 | 0.075 |
|  | AFz | 1.04.E-05 | 4.27.E-06 | 2.01.E-05 | 1.33.E-05 | 3.855^†^ | 0.001 |
|  | F1 | 1.21.E-05 | 4.68.E-06 | 1.68.E-05 | 8.62.E-06 | 2.645* | 0.016 |
|  | Fp1 | 9.90.E-06 | 2.96.E-06 | 2.95.E-05 | 3.28.E-05 | 3.317* | 0.004 |
|  | AF3 | 1.05.E-05 | 4.10.E-06 | 1.91.E-05 | 1.59.E-05 | 2.916* | 0.008 |
|  | F3 | 1.04.E-05 | 4.26.E-06 | 2.75.E-05 | 2.58.E-05 | 3.640* | 0.002 |
|  | F5 | 1.11.E-05 | 3.58.E-06 | 2.10.E-05 | 1.78.E-05 | 3.039* | 0.006 |
|  | FC5 | 1.17.E-05 | 5.11.E-06 | 2.15.E-05 | 1.51.E-05 | 3.394* | 0.003 |
|  | FC3 | 1.19.E-05 | 6.22.E-06 | 2.02.E-05 | 1.25.E-05 | 3.292* | 0.004 |
|  | C1 | 1.19.E-05 | 5.24.E-06 | 2.30.E-05 | 1.77.E-05 | 3.356* | 0.003 |
|  | F7 | 1.20.E-05 | 4.16.E-06 | 2.91.E-05 | 2.20.E-05 | 4.234^†^ | 0.001 |
|  | FT7 | 1.14.E-05 | 4.82.E-06 | 2.50.E-05 | 2.00.E-05 | 3.661* | 0.002 |
|  | C3 | 1.24.E-05 | 5.53.E-06 | 2.33.E-05 | 2.86.E-05 | 2.070 | 0.051 |
|  | CP1 | 1.23.E-05 | 4.05.E-06 | 2.03.E-05 | 1.62.E-05 | 2.665* | 0.015 |
|  | C5 | 1.24.E-05 | 5.44.E-06 | 2.35.E-05 | 3.58.E-05 | 1.702 | 0.099 |
|  | T3 | 1.09.E-05 | 3.76.E-06 | 2.19.E-05 | 1.87.E-05 | 3.204* | 0.004 |
|  | TP7 | 1.25.E-05 | 5.71.E-06 | 1.75.E-05 | 1.27.E-05 | 1.989 | 0.059 |
|  | CP5 | 1.35.E-05 | 5.88.E-06 | 2.56.E-05 | 2.00.E-05 | 3.196* | 0.004 |
|  | P5 | 1.34.E-05 | 5.27.E-06 | 2.55.E-05 | 1.67.E-05 | 3.843^†^ | 0.001 |
|  | P3 | 1.19.E-05 | 5.89.E-06 | 2.58.E-05 | 1.22.E-05 | 5.658^†^ | <0.001 |
|  | T5 | 1.28.E-05 | 6.03.E-06 | 2.56.E-05 | 2.76.E-05 | 2.517* | 0.021 |
|  | P1 | 1.12.E-05 | 3.76.E-06 | 1.97.E-05 | 1.29.E-05 | 3.517* | 0.002 |
|  | P9 | 1.32.E-05 | 5.92.E-06 | 3.04.E-05 | 4.27.E-05 | 2.231* | 0.037 |
|  | PO3 | 1.33.E-05 | 6.94.E-06 | 2.64.E-05 | 1.77.E-05 | 3.806* | 0.002 |
|  | Pz | 1.39.E-05 | 6.10.E-06 | 2.58.E-05 | 2.14.E-05 | 2.958* | 0.008 |
|  | O1 | 1.28.E-05 | 5.28.E-06 | 6.09.E-05 | 1.20.E-04 | 2.227* | 0.037 |
|  | POz | 1.31.E-05 | 6.08.E-06 | 2.58.E-05 | 2.84.E-05 | 2.433* | 0.025 |
|  | Oz | 1.32.E-05 | 6.22.E-06 | 4.34.E-05 | 6.24.E-05 | 2.689* | 0.015 |
|  | PO4 | 1.15.E-05 | 4.14.E-06 | 2.15.E-05 | 1.63.E-05 | 3.305* | 0.004 |
|  | O2 | 1.24.E-05 | 5.59.E-06 | 3.14.E-05 | 2.00.E-05 | 5.069^†^ | <0.001 |
|  | P2 | 1.05.E-05 | 4.84.E-06 | 2.00.E-05 | 2.63.E-05 | 1.983 | 0.059 |
|  | CP2 | 1.25.E-05 | 5.73.E-06 | 2.13.E-05 | 1.42.E-05 | 3.178* | 0.005 |
|  | P4 | 1.33.E-05 | 8.55.E-06 | 2.36.E-05 | 2.91.E-05 | 1.882 | 0.073 |
|  | P10 | 1.33.E-05 | 1.25.E-05 | 3.16.E-05 | 6.33.E-05 | 1.579 | 0.124 |
|  | T6 | 1.25.E-05 | 1.08.E-05 | 2.35.E-05 | 2.28.E-05 | 2.400* | 0.027 |
|  | P6 | 1.33.E-05 | 9.53.E-06 | 2.02.E-05 | 1.29.E-05 | 2.337* | 0.030 |
|  | CP6 | 1.40.E-05 | 8.89.E-06 | 2.55.E-05 | 2.55.E-05 | 2.362* | 0.029 |
|  | TP8 | 1.30.E-05 | 9.74.E-06 | 2.34.E-05 | 1.50.E-05 | 3.168* | 0.005 |
|  | C6 | 1.27.E-05 | 4.28.E-06 | 2.40.E-05 | 1.58.E-05 | 3.866^†^ | 0.001 |
|  | C4 | 1.46.E-05 | 8.17.E-06 | 2.44.E-05 | 1.31.E-05 | 3.494* | 0.003 |
|  | C2 | 1.30.E-05 | 5.32.E-06 | 2.57.E-05 | 1.40.E-05 | 4.700^†^ | <0.001 |
|  | T4 | 1.47.E-05 | 1.33.E-05 | 2.42.E-05 | 1.93.E-05 | 2.214* | 0.037 |
|  | FC4 | 1.27.E-05 | 3.99.E-06 | 2.42.E-05 | 1.40.E-05 | 4.361^†^ | <0.001 |
|  | FC2 | 1.31.E-05 | 5.01.E-06 | 1.66.E-05 | 9.17.E-06 | 1.832 | 0.078 |
|  | FT8 | 1.22.E-05 | 4.56.E-06 | 2.29.E-05 | 1.46.E-05 | 3.857^†^ | 0.001 |
|  | FC6 | 1.21.E-05 | 3.88.E-06 | 2.15.E-05 | 1.43.E-05 | 3.526* | 0.002 |
|  | F8 | 1.12.E-05 | 4.88.E-06 | 2.32.E-05 | 1.74.E-05 | 3.687* | 0.002 |
|  | F6 | 1.19.E-05 | 4.46.E-06 | 2.36.E-05 | 2.36.E-05 | 2.702* | 0.015 |
|  | F4 | 1.14.E-05 | 3.71.E-06 | 2.38.E-05 | 1.12.E-05 | 5.834^†^ | <0.001 |
|  | Cz | 1.53.E-05 | 1.50.E-05 | 1.58.E-05 | 9.42.E-06 | 0.152 | 0.880 |

| Resting-state Relative theta power (㎶) during the resting-state | AF4 | 0.443 | 0.131 | 0.520 | 0.113 | 2.530* | 0.027 |
| --- | --- | --- | --- | --- | --- | --- | --- |
|  | F2 | 0.462 | 0.149 | 0.567 | 0.119 | 3.141* | 0.010 |
|  | FCz | 0.511 | 0.181 | 0.659 | 0.139 | 3.714* | 0.003 |
|  | Fp2 | 0.449 | 0.117 | 0.536 | 0.119 | 2.995* | 0.015 |
|  | Fz | 0.467 | 0.155 | 0.563 | 0.131 | 2.689* | 0.024 |
|  | FC1 | 0.468 | 0.165 | 0.621 | 0.146 | 3.955* | 0.003 |
|  | AFz | 0.467 | 0.145 | 0.550 | 0.124 | 2.487* | 0.028 |
|  | F1 | 0.468 | 0.149 | 0.555 | 0.135 | 2.443* | 0.028 |
|  | Fp1 | 0.414 | 0.111 | 0.530 | 0.130 | 3.877* | 0.003 |
|  | AF3 | 0.429 | 0.129 | 0.507 | 0.140 | 2.331* | 0.034 |
|  | F3 | 0.416 | 0.117 | 0.540 | 0.135 | 3.964* | 0.003 |
|  | F5 | 0.412 | 0.118 | 0.491 | 0.128 | 2.590* | 0.025 |
|  | FC5 | 0.436 | 0.135 | 0.511 | 0.134 | 2.238* | 0.038 |
|  | FC3 | 0.453 | 0.136 | 0.534 | 0.138 | 2.372* | 0.031 |
|  | C1 | 0.472 | 0.157 | 0.586 | 0.130 | 3.184* | 0.010 |
|  | F7 | 0.432 | 0.128 | 0.539 | 0.139 | 3.212* | 0.010 |
|  | FT7 | 0.411 | 0.122 | 0.487 | 0.125 | 2.458* | 0.028 |
|  | C3 | 0.443 | 0.142 | 0.526 | 0.129 | 2.468* | 0.028 |
|  | CP1 | 0.491 | 0.167 | 0.570 | 0.109 | 2.265* | 0.038 |
|  | C5 | 0.437 | 0.126 | 0.501 | 0.128 | 2.009 | 0.056 |
|  | T3 | 0.433 | 0.133 | 0.565 | 0.155 | 3.691* | 0.003 |
|  | TP7 | 0.439 | 0.129 | 0.508 | 0.124 | 2.192* | 0.041 |
|  | CP5 | 0.445 | 0.139 | 0.531 | 0.123 | 2.642* | 0.025 |
|  | P5 | 0.463 | 0.153 | 0.549 | 0.111 | 2.591* | 0.025 |
|  | P3 | 0.469 | 0.160 | 0.553 | 0.114 | 2.448* | 0.028 |
|  | T5 | 0.476 | 0.171 | 0.566 | 0.130 | 2.380* | 0.031 |
|  | P1 | 0.462 | 0.149 | 0.553 | 0.104 | 2.880* | 0.016 |
|  | P9 | 0.480 | 0.163 | 0.571 | 0.131 | 2.488* | 0.028 |
|  | PO3 | 0.466 | 0.151 | 0.553 | 0.123 | 2.563* | 0.026 |
|  | Pz | 0.469 | 0.153 | 0.561 | 0.126 | 2.630* | 0.025 |
|  | O1 | 0.461 | 0.156 | 0.535 | 0.133 | 2.082 | 0.050 |
|  | POz | 0.469 | 0.150 | 0.546 | 0.127 | 2.242* | 0.038 |
|  | Oz | 0.459 | 0.149 | 0.529 | 0.135 | 2.006 | 0.056 |
|  | PO4 | 0.466 | 0.145 | 0.549 | 0.129 | 2.456* | 0.028 |
|  | O2 | 0.462 | 0.156 | 0.535 | 0.125 | 2.092 | 0.050 |
|  | P2 | 0.468 | 0.143 | 0.555 | 0.126 | 2.609* | 0.025 |
|  | CP2 | 0.442 | 0.151 | 0.567 | 0.115 | 3.738* | 0.003 |
|  | P4 | 0.465 | 0.140 | 0.544 | 0.151 | 2.187* | 0.041 |
|  | P10 | 0.469 | 0.164 | 0.539 | 0.126 | 1.935 | 0.063 |
|  | T6 | 0.477 | 0.165 | 0.530 | 0.145 | 1.376 | 0.177 |
|  | P6 | 0.470 | 0.148 | 0.544 | 0.142 | 2.065 | 0.051 |
|  | CP6 | 0.467 | 0.150 | 0.536 | 0.132 | 1.969 | 0.060 |
|  | TP8 | 0.471 | 0.152 | 0.534 | 0.135 | 1.774 | 0.085 |
|  | C6 | 0.461 | 0.147 | 0.518 | 0.126 | 1.671 | 0.103 |
|  | C4 | 0.447 | 0.144 | 0.541 | 0.112 | 2.954* | 0.015 |
|  | C2 | 0.450 | 0.150 | 0.594 | 0.127 | 4.175* | 0.003 |
|  | T4 | 0.479 | 0.136 | 0.584 | 0.122 | 3.272* | 0.010 |
|  | FC4 | 0.459 | 0.147 | 0.558 | 0.123 | 2.962* | 0.015 |
|  | FC2 | 0.457 | 0.160 | 0.626 | 0.137 | 4.574^†^ | 0.001 |
|  | FT8 | 0.452 | 0.138 | 0.523 | 0.120 | 2.234* | 0.038 |
|  | FC6 | 0.448 | 0.143 | 0.537 | 0.119 | 2.740* | 0.022 |
|  | F8 | 0.461 | 0.132 | 0.564 | 0.133 | 3.150* | 0.010 |
|  | F6 | 0.438 | 0.132 | 0.521 | 0.107 | 2.776* | 0.021 |
|  | F4 | 0.459 | 0.130 | 0.551 | 0.124 | 2.926* | 0.015 |
|  | Cz | 0.476 | 0.178 | 0.637 | 0.158 | 3.865* | 0.003 |
| MA test  relative theta power (㎶) during the MA test | AF4 | 0.480 | 0.188 | 0.436 | 0.113 | -1.138 | 0.834 |
|  | F2 | 0.524 | 0.217 | 0.475 | 0.140 | -1.093 | 0.834 |
|  | FCz | 0.549 | 0.220 | 0.532 | 0.159 | -0.365 | 0.885 |
|  | Fp2 | 0.482 | 0.187 | 0.469 | 0.122 | -0.325 | 0.885 |
|  | Fz | 0.555 | 0.258 | 0.519 | 0.136 | -0.709 | 0.834 |
|  | FC1 | 0.505 | 0.219 | 0.509 | 0.147 | 0.099 | 0.938 |
|  | AFz | 0.526 | 0.211 | 0.468 | 0.135 | -1.317 | 0.834 |
|  | F1 | 0.510 | 0.189 | 0.476 | 0.143 | -0.821 | 0.834 |
|  | Fp1 | 0.481 | 0.183 | 0.471 | 0.114 | -0.265 | 0.885 |
|  | AF3 | 0.476 | 0.174 | 0.440 | 0.119 | -0.976 | 0.834 |
|  | F3 | 0.490 | 0.223 | 0.482 | 0.112 | -0.185 | 0.918 |
|  | F5 | 0.446 | 0.131 | 0.418 | 0.099 | -0.996 | 0.834 |
|  | FC5 | 0.460 | 0.180 | 0.420 | 0.109 | -1.105 | 0.834 |
|  | FC3 | 0.455 | 0.171 | 0.445 | 0.121 | -0.271 | 0.885 |
|  | C1 | 0.460 | 0.129 | 0.483 | 0.134 | 0.719 | 0.834 |
|  | F7 | 0.495 | 0.200 | 0.470 | 0.096 | -0.652 | 0.834 |
|  | FT7 | 0.453 | 0.169 | 0.409 | 0.093 | -1.312 | 0.834 |
|  | C3 | 0.454 | 0.185 | 0.434 | 0.108 | -0.538 | 0.885 |
|  | CP1 | 0.484 | 0.152 | 0.487 | 0.122 | 0.076 | 0.940 |
|  | C5 | 0.449 | 0.144 | 0.410 | 0.116 | -1.207 | 0.834 |
|  | T3 | 0.489 | 0.201 | 0.461 | 0.119 | -0.700 | 0.834 |
|  | TP7 | 0.462 | 0.194 | 0.418 | 0.112 | -1.125 | 0.834 |
|  | CP5 | 0.478 | 0.224 | 0.442 | 0.128 | -0.811 | 0.834 |
|  | P5 | 0.483 | 0.188 | 0.440 | 0.117 | -1.128 | 0.834 |
|  | P3 | 0.464 | 0.139 | 0.460 | 0.130 | -0.107 | 0.938 |
|  | T5 | 0.479 | 0.164 | 0.424 | 0.118 | -1.562 | 0.834 |
|  | P1 | 0.490 | 0.195 | 0.464 | 0.128 | -0.620 | 0.834 |
|  | P9 | 0.491 | 0.205 | 0.423 | 0.099 | -1.732 | 0.834 |
|  | PO3 | 0.485 | 0.208 | 0.468 | 0.124 | -0.396 | 0.885 |
|  | Pz | 0.491 | 0.151 | 0.481 | 0.130 | -0.279 | 0.885 |
|  | O1 | 0.458 | 0.195 | 0.420 | 0.127 | -0.956 | 0.834 |
|  | POz | 0.488 | 0.203 | 0.450 | 0.128 | -0.917 | 0.834 |
|  | Oz | 0.472 | 0.237 | 0.423 | 0.126 | -1.052 | 0.834 |
|  | PO4 | 0.497 | 0.229 | 0.450 | 0.118 | -1.042 | 0.834 |
|  | O2 | 0.469 | 0.236 | 0.404 | 0.117 | -1.425 | 0.834 |
|  | P2 | 0.486 | 0.180 | 0.453 | 0.117 | -0.875 | 0.834 |
|  | CP2 | 0.470 | 0.166 | 0.485 | 0.099 | 0.465 | 0.885 |
|  | P4 | 0.482 | 0.225 | 0.452 | 0.112 | -0.701 | 0.834 |
|  | P10 | 0.450 | 0.150 | 0.430 | 0.106 | -0.614 | 0.834 |
|  | T6 | 0.473 | 0.198 | 0.414 | 0.117 | -1.466 | 0.834 |
|  | P6 | 0.453 | 0.139 | 0.449 | 0.109 | -0.145 | 0.934 |
|  | CP6 | 0.482 | 0.228 | 0.454 | 0.108 | -0.651 | 0.834 |
|  | TP8 | 0.460 | 0.209 | 0.442 | 0.101 | -0.444 | 0.885 |
|  | C6 | 0.458 | 0.206 | 0.427 | 0.099 | -0.781 | 0.834 |
|  | C4 | 0.454 | 0.128 | 0.445 | 0.107 | -0.329 | 0.885 |
|  | C2 | 0.484 | 0.177 | 0.470 | 0.120 | -0.388 | 0.885 |
|  | T4 | 0.476 | 0.195 | 0.458 | 0.115 | -0.447 | 0.885 |
|  | FC4 | 0.488 | 0.219 | 0.438 | 0.124 | -1.140 | 0.834 |
|  | FC2 | 0.515 | 0.226 | 0.493 | 0.151 | -0.462 | 0.885 |
|  | FT8 | 0.462 | 0.204 | 0.409 | 0.096 | -1.357 | 0.834 |
|  | FC6 | 0.458 | 0.128 | 0.416 | 0.107 | -1.426 | 0.834 |
|  | F8 | 0.499 | 0.205 | 0.483 | 0.086 | -0.428 | 0.885 |
|  | F6 | 0.467 | 0.167 | 0.418 | 0.094 | -1.482 | 0.834 |
|  | F4 | 0.514 | 0.248 | 0.481 | 0.111 | -0.720 | 0.834 |
|  | Cz | 0.517 | 0.217 | 0.525 | 0.135 | 0.187 | 0.918 |

PD: Panic disorder, HC: Healthy control, MA: Mental arithmetic, SD: Standard deviation, FDR: False Discovery Rate, MI: Modulation index. *p≤0.05, †p≤0.001.

**Supplementary Table 2.** Pearson’s correlation coefficients between the EEG measures and clinical characteristics across PD patients and HCs

| Lead | TGC during the RS  condition | | TGC during the MA condition | | TGC during the MA condition | | Relative theta power  during the RS  condition | | Relative theta power  during the MA  condition | | |
| --- | --- | --- | --- | --- | --- | --- | --- | --- | --- | --- | --- |
|  | STAI-S | | STAI-S | | MA Score | | PSS | | | MA Score | |
|  | R | *p-*_FDR_ | R | *p-*_FDR_ | R | *p-*_FDR_ | R | *p-*_FDR_ | | R | *p-*_FDR_ |
| AF4 | -0.408* | 0.034 | -0.272 | 0.067 | 0.192 | 0.304 | -0.266 | 0.079 | | 0.288 | 0.070 |
| F2 | -0.175 | 0.275 | -0.281 | 0.060 | 0.148 | 0.386 | -0.196 | 0.190 | | 0.346* | 0.034 |
| FCz | -0.147 | 0.331 | -0.260 | 0.072 | 0.420* | 0.026 | -0.272 | 0.079 | | 0.415* | 0.014 |
| Fp2 | -0.430* | 0.034 | -0.258 | 0.072 | 0.120 | 0.495 | -0.334* | 0.041 | | 0.234 | 0.105 |
| Fz | -0.215 | 0.195 | -0.246 | 0.088 | 0.179 | 0.324 | -0.254 | 0.090 | | 0.238 | 0.100 |
| FC1 | -0.106 | 0.460 | -0.237 | 0.098 | 0.491* | 0.004 | -0.262 | 0.081 | | 0.444* | 0.011 |
| AFz | -0.173 | 0.275 | -0.297* | 0.050 | 0.248 | 0.185 | -0.287 | 0.068 | | 0.200 | 0.153 |
| F1 | -0.267 | 0.139 | -0.300* | 0.050 | 0.231 | 0.232 | -0.325* | 0.041 | | 0.284 | 0.070 |
| Fp1 | -0.269 | 0.139 | -0.268 | 0.067 | 0.170 | 0.331 | -0.346* | 0.041 | | 0.212 | 0.133 |
| AF3 | -0.272 | 0.139 | -0.293 | 0.053 | 0.160 | 0.349 | -0.276 | 0.077 | | 0.239 | 0.100 |
| F3 | -0.348 | 0.091 | -0.266 | 0.067 | 0.107 | 0.544 | -0.347* | 0.041 | | 0.281 | 0.070 |
| F5 | -0.230 | 0.182 | -0.427* | 0.006 | 0.219 | 0.241 | -0.279 | 0.077 | | 0.168 | 0.216 |
| FC5 | -0.247 | 0.155 | -0.448* | 0.006 | 0.305 | 0.139 | -0.266 | 0.079 | | 0.256 | 0.083 |
| FC3 | -0.281 | 0.139 | -0.303* | 0.050 | 0.314 | 0.139 | -0.227 | 0.128 | | 0.372* | 0.027 |
| C1 | -0.251 | 0.155 | -0.401* | 0.008 | 0.224 | 0.239 | -0.167 | 0.268 | | 0.353* | 0.034 |
| F7 | -0.333 | 0.107 | -0.430* | 0.006 | 0.293 | 0.150 | -0.356* | 0.041 | | 0.292 | 0.069 |
| FT7 | -0.211 | 0.195 | -0.443* | 0.006 | 0.277 | 0.151 | -0.265 | 0.079 | | 0.192 | 0.165 |
| C3 | -0.304 | 0.129 | -0.310* | 0.046 | 0.276 | 0.151 | -0.230 | 0.124 | | 0.259 | 0.083 |
| CP1 | -0.162 | 0.300 | -0.341* | 0.027 | 0.125 | 0.481 | -0.157 | 0.286 | | 0.363* | 0.031 |
| C5 | -0.145 | 0.331 | -0.179 | 0.226 | 0.260 | 0.178 | -0.156 | 0.286 | | 0.169 | 0.216 |
| T3 | -0.240 | 0.162 | -0.266 | 0.067 | 0.180 | 0.324 | -0.326* | 0.041 | | 0.337* | 0.036 |
| TP7 | -0.254 | 0.155 | -0.167 | 0.239 | 0.091 | 0.584 | -0.156 | 0.286 | | 0.206 | 0.142 |
| CP5 | -0.179 | 0.270 | -0.289 | 0.055 | 0.304 | 0.139 | -0.158 | 0.286 | | 0.283 | 0.070 |
| P5 | -0.194 | 0.231 | -0.283 | 0.060 | 0.318 | 0.139 | -0.106 | 0.454 | | 0.268 | 0.075 |
| P3 | -0.153 | 0.321 | -0.470* | 0.006 | 0.333 | 0.139 | -0.134 | 0.361 | | 0.265 | 0.077 |
| T5 | -0.067 | 0.632 | -0.149 | 0.287 | 0.055 | 0.758 | -0.127 | 0.377 | | 0.275 | 0.072 |
| P1 | -0.181 | 0.270 | -0.405* | 0.008 | 0.289 | 0.150 | -0.254 | 0.090 | | 0.376 | 0.027 |
| P9 | -0.158 | 0.311 | -0.168 | 0.239 | 0.000 | 0.998 | -0.128 | 0.377 | | 0.279 | 0.070 |
| PO3 | -0.301 | 0.129 | -0.370* | 0.014 | 0.229 | 0.232 | -0.318* | 0.045 | | 0.389 | 0.025 |
| Pz | -0.207 | 0.200 | -0.268 | 0.067 | 0.187 | 0.309 | -0.327* | 0.041 | | 0.257 | 0.083 |
| O1 | -0.226 | 0.182 | -0.150 | 0.287 | 0.140 | 0.414 | -0.287 | 0.068 | | 0.383* | 0.025 |
| POz | -0.218 | 0.190 | -0.173 | 0.236 | 0.200 | 0.292 | -0.327* | 0.041 | | 0.336* | 0.036 |
| Oz | -0.212 | 0.195 | -0.154 | 0.279 | 0.118 | 0.495 | -0.330* | 0.041 | | 0.347* | 0.034 |
| PO4 | -0.172 | 0.275 | -0.139 | 0.316 | 0.027 | 0.888 | -0.341* | 0.041 | | 0.252 | 0.087 |
| O2 | -0.165 | 0.294 | -0.327* | 0.036 | 0.277 | 0.151 | -0.305 | 0.056 | | 0.279 | 0.070 |
| P2 | -0.137 | 0.350 | -0.119 | 0.383 | 0.006 | 0.998 | -0.340* | 0.041 | | 0.179 | 0.194 |
| CP2 | -0.221 | 0.189 | -0.127 | 0.359 | 0.214 | 0.248 | -0.396* | 0.041 | | 0.465* | 0.009 |
| P4 | -0.144 | 0.331 | -0.089 | 0.508 | 0.030 | 0.883 | -0.214 | 0.150 | | 0.306 | 0.059 |
| P10 | -0.297 | 0.129 | -0.170 | 0.239 | 0.099 | 0.578 | -0.221 | 0.137 | | 0.278 | 0.070 |
| T6 | -0.203 | 0.207 | -0.259 | 0.072 | 0.175 | 0.327 | -0.095 | 0.475 | | 0.224 | 0.117 |
| P6 | -0.247 | 0.155 | -0.238 | 0.098 | -0.049 | 0.784 | -0.185 | 0.215 | | 0.328* | 0.039 |
| CP6 | -0.226 | 0.182 | -0.174 | 0.236 | 0.084 | 0.602 | -0.143 | 0.331 | | 0.247 | 0.090 |
| TP8 | -0.120 | 0.419 | -0.374* | 0.014 | 0.091 | 0.584 | -0.098 | 0.467 | | 0.269 | 0.075 |
| C6 | -0.118 | 0.423 | -0.415* | 0.008 | 0.248 | 0.185 | -0.104 | 0.459 | | 0.272 | 0.074 |
| C4 | -0.243 | 0.158 | -0.313* | 0.046 | 0.341 | 0.139 | -0.298 | 0.062 | | 0.344* | 0.034 |
| C2 | -0.058 | 0.660 | -0.277 | 0.063 | 0.187 | 0.309 | -0.377* | 0.041 | | 0.421* | 0.014 |
| T4 | -0.079 | 0.576 | -0.186 | 0.210 | 0.089 | 0.584 | -0.290 | 0.068 | | 0.293 | 0.069 |
| FC4 | -0.065 | 0.634 | -0.400* | 0.008 | 0.255 | 0.184 | -0.209 | 0.158 | | 0.420* | 0.014 |
| FC2 | -0.150 | 0.326 | -0.298* | 0.050 | 0.218 | 0.241 | -0.365* | 0.041 | | 0.472* | 0.009 |
| FT8 | -0.294 | 0.129 | -0.435* | 0.006 | 0.172 | 0.331 | -0.117 | 0.410 | | 0.217 | 0.129 |
| FC6 | -0.094 | 0.511 | -0.408* | 0.008 | 0.194 | 0.304 | -0.185 | 0.215 | | 0.294 | 0.069 |
| F8 | -0.303 | 0.129 | -0.372* | 0.014 | 0.265 | 0.172 | -0.269 | 0.079 | | 0.226 | 0.116 |
| F6 | -0.357 | 0.091 | -0.387* | 0.011 | 0.164 | 0.349 | -0.231 | 0.124 | | 0.247 | 0.090 |
| F4 | -0.273 | 0.139 | -0.214 | 0.140 | 0.150 | 0.385 | -0.249 | 0.094 | | 0.214 | 0.133 |
| Cz | -0.257 | 0.155 | -0.070 | 0.596 | 0.001 | 0.998 | -0.430* | 0.031 | | 0.351* | 0.034 |

Values are Pearson’s correlation coefficients. *p≤0.05. EEG: electroencephalography, PD: Panic disorder, HC: Healthy control, RS: Resting State MA: Mental Arithmetic, SD: Standard deviation, FDR: False Discovery Rate, TGC: Theta-Gamma Coupling, STAI-S: State-Trait Anxiety Inventory, PSS: Perceived Stress Scale.

**Supplementary Table 3.** Pearson’s correlation coefficients between the relative theta power and HAMA scores in PD patients

| Lead | Relative theta power during the RS condition | |
| --- | --- | --- |
|  | HAMA | |
|  | R | *p-*_FDR_ |
| AF4 | -0.460328164* | 0.011316412 |
| F2 | -0.398993456* | 0.020878101 |
| FCz | -0.446661719* | 0.012508139 |
| Fp2 | -0.452465503* | 0.011749719 |
| Fz | -0.470174684* | 0.011316412 |
| FC1 | -0.373022332* | 0.030124335 |
| AFz | -0.463495057* | 0.011316412 |
| F1 | -0.46760822* | 0.011316412 |
| Fp1 | -0.45357391* | 0.011749719 |
| AF3 | -0.436706953* | 0.013247473 |
| F3 | -0.402054449* | 0.020584473 |
| F5 | -0.400370007* | 0.020783062 |
| FC5 | -0.430947703* | 0.013563088 |
| FC3 | -0.438102441* | 0.013247473 |
| C1 | -0.37232621* | 0.030124335 |
| F7 | -0.494660272* | 0.010706575 |
| FT7 | -0.455386884* | 0.011749719 |
| C3 | -0.452916372* | 0.011749719 |
| CP1 | -0.462143155* | 0.011316412 |
| C5 | -0.532941473* | 0.009561358 |
| T3 | -0.56055235* | 0.006955465 |
| TP7 | -0.553164191* | 0.006955465 |
| CP5 | -0.519503889* | 0.010389692 |
| P5 | -0.468901198* | 0.011316412 |
| P3 | -0.462000367* | 0.011316412 |
| T5 | -0.445142411* | 0.012508139 |
| P1 | -0.488173156* | 0.010706575 |
| P9 | -0.480103381* | 0.011316412 |
| PO3 | -0.551023871* | 0.006955465 |
| Pz | -0.56397474* | 0.006955465 |
| O1 | -0.437719799* | 0.013247473 |
| POz | -0.580611762* | 0.006955465 |
| Oz | -0.524022806* | 0.010389692 |
| PO4 | -0.560373637* | 0.006955465 |
| O2 | -0.465787976* | 0.011316412 |
| P2 | -0.510473622* | 0.010706575 |
| CP2 | -0.500519517* | 0.010706575 |
| P4 | -0.499459112* | 0.010706575 |
| P10 | -0.489178926* | 0.010706575 |
| T6 | -0.468983211* | 0.011316412 |
| P6 | -0.491063264* | 0.010706575 |
| CP6 | -0.465817504* | 0.011316412 |
| A2 | -0.446295949* | 0.012508139 |
| TP8 | -0.461127888* | 0.011316412 |
| C6 | -0.435635381* | 0.013247473 |
| C4 | -0.414586271* | 0.017545243 |
| C2 | -0.387149113* | 0.024556841 |
| T4 | -0.508966885* | 0.010706575 |
| FC4 | -0.413139352* | 0.01763732 |
| FC2 | -0.437372491* | 0.013247473 |
| FT8 | -0.402383394* | 0.020584473 |
| FC6 | -0.396260787* | 0.021468846 |
| F8 | -0.433141088* | 0.013303467 |
| F6 | -0.434551007* | 0.013247473 |
| F4 | -0.428905002* | 0.013791867 |
| Cz | -0.498891966* | 0.010706575 |

Values are Pearson’s correlation coefficients. *p≤0.05. RS: Resting-state, HAMA: Hamilton Rating Scale for Anxiety, PD: Panic disorder, FDR: False Discovery Rate.

**Supplementary Table 4.** General Demographic and Medication Details for PD patients

| No | Age | Sex | Medication (mg/d) |
| --- | --- | --- | --- |
| 1 | 24 | M | Alprazolam (0.25 mg) |
| Daily dose of BDZ^a^ | | | 0.5 |
| 2 | 29 | M | Clonazepam (1 mg), Alprazolam (1 mg), Paroxetine Hydrochloride (37.5 mg) |
| Daily dose of BDZ^a^ | | | 4.67 |
| 3 | 34 | M | Alprazolam (0.25 mg), Paroxetine Hydrochloride (12.5 mg),Propranolol Hydrochloride (20 mg), |
| Daily dose of BDZ^a^ | | | 0.5 |
| 4 | 24 | F | Escitalopram Oxalate (5 mg) |
| Daily dose of BDZ^a^ | | | 0 |
| 5 | 26 | F |  |
| Daily dose of BDZ^a^ | | | 0 |
| 6 | 26 | F | Clonazepam (1 mg), Paroxetine Hydrochloride (12.5 mg) |
| Daily dose of BDZ^a^ | | | 2.67 |
| 7 | 30 | F | Alprazolam (0.75 mg), Escitalopram Oxalate (5 mg) |
| Daily dose of BDZ^a^ | | | 1.5 |
| 8 | 24 | M | Clonazepam (1.5 mg) |
| Daily dose of BDZ^a^ | | | 4 |
| 9 | 26 | F | Clonazepam (3.75 mg), Escitalopram Oxalate (5 mg) |
| Daily dose of BDZ^a^ | | | 10 |
| 10 | 25 | M | Alprazolam (0.5 mg) |
| Daily dose of BDZ^a^ | | | 1 |
| 11 | 23 | F | Alprazolam (0.625 mg), Paroxetine Hydrochloride (12.5 mg) |
| Daily dose of BDZ^a^ | | | 1.25 |
| 12 | 22 | F | Sertraline (25 mg), Trazodone Hydrochloride (25 mg) |
| Daily dose of BDZ^a^ | | | 0 |
| 13 | 31 | F | Alprazolam (0.125 mg), Clonazepam (0.25 mg), Escitalopram Oxalate (5 mg) |
| Daily dose of BDZ^a^ | | | 1.75 |
| 14 | 25 | M | Etizolam (1.5 mg), Sertraline (50 mg) |
| Daily dose of BDZ^a^ | | | 1 |
| 15 | 27 | F | Alprazolam (1 mg),Escitalopram Oxalate (5 mg) |
| Daily dose of BDZ^a^ | | | 2 |
| 16 | 20 | F | Clonazepam (0.75 mg),Escitalopram Oxalate (10 mg) |
| Daily dose of BDZ^a^ | | | 2 |
| 17 | 27 | F | Alprazolam (0.75 mg), Escitalopram Oxalate (5 mg) |
| Daily dose of BDZ^a^ | | | 1.5 |
| 18 | 23 | F | Alprazolam (0.75 mg), Escitalopram Oxalate (10 mg) |
| Daily dose of BDZ^a^ | | | 1.5 |
| 19 | 31 | F | Clonazepam (0.75 mg), Escitalopram Oxalate (5 mg) |
| Daily dose of BDZ^a^ | | | 2 |
| 20 | 27 | M | Clonazepam (0.5 mg), Paroxetine Hydrochloride (25 mg) |
| Daily dose of BDZ^a^ | | | 1.34 |
| 21 | 23 | F | Alprazolam (0.25 mg) |
| Daily dose of BDZ^a^ | | | 0.5 |
| 22 | 26 | F | Alprazolam (0.75 mg), Lorazepam (0.5 mg), Sertraline (100 mg), Propranolol Hydrochloride (20 mg) |
| Daily dose of BDZ^a^ | | | 2 |
| 23 | 32 | F | Alprazolam (0.375 mg), Escitalopram Oxalate (5 mg) |
| Daily dose of BDZ^a^ | | | 0.75 |
| 24 | 20 | F | Alprazolam (0.125 mg), Escitalopram Oxalate (5 mg) |
| Daily dose of BDZ^a^ | | | 0.25 |
| 25 | 41 | M | Sodium Tianeptine (25 mg), Escitalopram Oxalate (10 mg) |
| Daily dose of BDZ^a^ | | | 0 |
| 26 | 24 | M | Alprazolam (0.75 mg), Escitalopram Oxalate (5 mg) |
| Daily dose of BDZ^a^ | | | 1.5 |
| 27 | 25 | M | Clonazepam (0.5 mg), Alprazolam (0.5 mg), Zolpidem Tartrate (10 mg), Paroxetine Hydrochloride (25 mg), Venlafaxine (75 mg), |
| Daily dose of BDZ^a^ | | | 2.34 |
| 28 | 25 | F | Alprazolam (0.5 mg), Clonazepam (1 mg), Bortioxetine (5 mg), Duloxetine (60 mg), Fluoxetine (20 mg), Aripiprazole (2 mg), Propranolol Hydrochloride (40 mg) |
| Daily dose of BDZ^a^ | | | 3.67 |
| 29 | 24 | M | Alprazolam (1.25 mg), Buspirone Hydrochloride (15 mg), Escitalopram Oxalate (20 mg), Quetiapine (25 mg) |
| Daily dose of BDZ^a^ | | | 2.5 |
| 30 | 21 | M | Alprazolam (0.25 mg), Escitalopram Oxalate (5 mg) |
| Daily dose of BDZ^a^ | | | 2.5 |
| 31 | 28 | M | Alprazolam (1.5 mg), Clonazepam (1.5 mg), Paroxetine Hydrochloride (40 mg), Mirtazapine (7.5 mg), Propranolol Hydrochloride (30 mg), |
| Daily dose of BDZ^a^ | | | 7 |
| 32 | 24 | M | Alprazolam (0.5 mg),Escitalopram Oxalate (5 mg) |
| Daily dose of BDZ^a^ | | | 1 |
| 33 | 33 | F | Buspirone Hydrochloride (20 mg) |
| Daily dose of BDZ^a^ | | | 0 |
| 34 | 21 | F | Alprazolam (0.25 mg) |
| Daily dose of BDZ^a^ | | | 0.5 |

PD: Panic disorder. Antidepressants (escitalopram, fluoxetine, paroxetine, sertraline, duloxetine, venlafaxine, mirtazapine, bortioxetine, trazodone, sodium tianeptine); Benzodiazepines (alprazolam, clonazepam, etizolam, lorazepam, zolpidem); Non-benzodiazepine anxiolytics (buspirone); Antipsychotics (aripiprazole, quetiapine); Other medication (propranolol hydrochloride). ^a^ Data presented as oral lorazepam-equivalent dose for benzodiazepines. Benzodiazepine doses were calculated and transformed into lorazepam equivalents. Lorazepam 1mg equivalent doses: diazepam 5 mg, clonazepam 0.375 mg, alprazolam 0.5 mg, etizolam 1 mg.

**Supplementary Table 5.** Pearson's correlation coefficient for clinical and medication data (Spearman rho)

| Variables | HAMA | HAMD | STAI-S | PSS | MA score | BDZ dose |
| --- | --- | --- | --- | --- | --- | --- |
| HAMA | 1 |  |  |  |  |  |
| HAMD | 0.90** | 1 |  |  |  |  |
| STAI-S | 0.62** | 0.62** | 1 |  |  |  |
| PSS | 0.57** | 0.67** | 0.69** | 1 |  |  |
| MA score | -0.66** | -0.58** | -0.52** | -0.47** | 1 |  |
| BDZ dose | 0.26 | 0.36* | 0.38* | 0.44** | -0.05* | 1 |

PD, panic disorder; HC, healthy controls; HAM-A, Hamilton Rating Scale for Anxiety; HAM-D, Hamilton Rating Scale for Depression; ASI, Anxiety Sensitivity Index; PSS, Perceived Stress Scale. * p <0.05, ** p <0.001.

**Supplementary Table 6.** Pearson’s correlation coefficients between EEG measures and daily dose of BDZ in PD patients

| Lead | TGC during RS | | TGC during MA | | Relative theta power  during RS | | Relative theta power  during MA | |
| --- | --- | --- | --- | --- | --- | --- | --- | --- |
|  | Daily dose of BDZ^a^ | | | | | | | |
|  | R | *p-*_FDR_ | R | *p-*_FDR_ | R | *p-*_FDR_ | R | *p-*_FDR_ |
| AF4 | -0.189 | 0.981 | 0.068 | 0.988 | -0.024 | 0.990 | 0.021 | 0.997 |
| F2 | -0.090 | 0.981 | 0.105 | 0.988 | 0.047 | 0.990 | 0.070 | 0.997 |
| FCz | 0.036 | 0.981 | 0.047 | 0.988 | 0.019 | 0.990 | 0.125 | 0.997 |
| Fp2 | 0.126 | 0.981 | -0.106 | 0.988 | -0.067 | 0.990 | -0.021 | 0.997 |
| Fz | 0.135 | 0.981 | 0.030 | 0.988 | -0.067 | 0.990 | 0.013 | 0.997 |
| FC1 | 0.095 | 0.981 | 0.077 | 0.988 | -0.089 | 0.990 | -0.069 | 0.997 |
| AFz | 0.057 | 0.981 | 0.114 | 0.988 | -0.076 | 0.990 | 0.041 | 0.997 |
| F1 | 0.043 | 0.981 | -0.003 | 0.988 | -0.064 | 0.990 | 0.011 | 0.997 |
| Fp1 | -0.021 | 0.981 | -0.005 | 0.988 | -0.018 | 0.990 | -0.026 | 0.997 |
| AF3 | -0.043 | 0.981 | 0.102 | 0.988 | -0.023 | 0.990 | 0.025 | 0.997 |
| F3 | 0.178 | 0.981 | 0.061 | 0.988 | -0.015 | 0.990 | 0.009 | 0.997 |
| F5 | -0.105 | 0.981 | -0.155 | 0.988 | -0.010 | 0.990 | 0.089 | 0.997 |
| FC5 | -0.031 | 0.981 | 0.261 | 0.988 | -0.085 | 0.990 | 0.057 | 0.997 |
| FC3 | -0.193 | 0.981 | 0.305 | 0.988 | -0.059 | 0.990 | -0.043 | 0.997 |
| C1 | -0.082 | 0.981 | 0.021 | 0.988 | -0.041 | 0.990 | -0.049 | 0.997 |
| F7 | 0.149 | 0.981 | -0.030 | 0.988 | -0.088 | 0.990 | -0.008 | 0.997 |
| FT7 | -0.125 | 0.981 | 0.259 | 0.988 | -0.098 | 0.990 | 0.115 | 0.997 |
| C3 | 0.008 | 0.981 | 0.153 | 0.988 | -0.100 | 0.990 | -0.030 | 0.997 |
| CP1 | -0.071 | 0.981 | 0.054 | 0.988 | -0.004 | 0.990 | -0.090 | 0.997 |
| C5 | -0.252 | 0.981 | 0.217 | 0.988 | -0.038 | 0.990 | 0.051 | 0.997 |
| T3 | 0.075 | 0.981 | 0.142 | 0.988 | -0.170 | 0.990 | -0.114 | 0.997 |
| TP7 | -0.054 | 0.981 | 0.209 | 0.988 | -0.022 | 0.990 | -0.003 | 0.997 |
| CP5 | 0.074 | 0.981 | 0.015 | 0.988 | -0.021 | 0.990 | -0.028 | 0.997 |
| P5 | -0.008 | 0.981 | 0.056 | 0.988 | 0.113 | 0.990 | -0.012 | 0.997 |
| P3 | 0.080 | 0.981 | 0.044 | 0.988 | 0.056 | 0.990 | 0.009 | 0.997 |
| A1 | 0.050 | 0.981 | 0.204 | 0.988 | 0.073 | 0.990 | 0.006 | 0.997 |
| T5 | -0.145 | 0.981 | 0.008 | 0.988 | 0.146 | 0.990 | -0.048 | 0.997 |
| P1 | 0.080 | 0.981 | 0.123 | 0.988 | -0.079 | 0.990 | -0.025 | 0.997 |
| P9 | -0.141 | 0.981 | 0.103 | 0.988 | 0.256 | 0.990 | 0.007 | 0.997 |
| PO3 | -0.116 | 0.981 | 0.244 | 0.988 | -0.200 | 0.990 | 0.057 | 0.997 |
| Pz | -0.004 | 0.981 | 0.105 | 0.988 | -0.187 | 0.990 | -0.013 | 0.997 |
| O1 | 0.025 | 0.981 | -0.189 | 0.988 | -0.121 | 0.990 | 0.006 | 0.997 |
| POz | -0.090 | 0.981 | 0.027 | 0.988 | -0.177 | 0.990 | -0.001 | 0.997 |
| Oz | -0.018 | 0.981 | -0.114 | 0.988 | -0.137 | 0.990 | 0.018 | 0.997 |
| PO4 | 0.168 | 0.981 | -0.003 | 0.988 | -0.185 | 0.990 | 0.024 | 0.997 |
| O2 | 0.188 | 0.981 | 0.053 | 0.988 | -0.143 | 0.990 | 0.015 | 0.997 |
| P2 | 0.237 | 0.981 | 0.213 | 0.988 | -0.129 | 0.990 | 0.020 | 0.997 |
| CP2 | 0.193 | 0.981 | 0.114 | 0.988 | -0.055 | 0.990 | 0.039 | 0.997 |
| P4 | 0.144 | 0.981 | 0.161 | 0.988 | -0.107 | 0.990 | -0.003 | 0.997 |
| P10 | -0.044 | 0.981 | 0.035 | 0.988 | -0.031 | 0.990 | 0.024 | 0.997 |
| T6 | 0.178 | 0.981 | 0.163 | 0.988 | -0.002 | 0.990 | -0.012 | 0.997 |
| P6 | 0.114 | 0.981 | 0.097 | 0.988 | -0.103 | 0.990 | 0.036 | 0.997 |
| CP6 | 0.012 | 0.981 | 0.015 | 0.988 | -0.071 | 0.990 | 0.024 | 0.997 |
| A2 | -0.052 | 0.981 | 0.097 | 0.988 | 0.179 | 0.990 | 0.046 | 0.997 |
| TP8 | 0.120 | 0.981 | 0.192 | 0.988 | 0.064 | 0.990 | 0.028 | 0.997 |
| C6 | -0.079 | 0.981 | 0.234 | 0.988 | 0.104 | 0.990 | 0.056 | 0.997 |
| C4 | 0.026 | 0.981 | 0.287 | 0.988 | -0.020 | 0.990 | -0.025 | 0.997 |
| C2 | 0.109 | 0.981 | 0.200 | 0.988 | -0.031 | 0.990 | 0.013 | 0.997 |
| T4 | 0.091 | 0.981 | 0.087 | 0.988 | -0.092 | 0.990 | -0.123 | 0.997 |
| FC4 | -0.237 | 0.981 | 0.188 | 0.988 | 0.174 | 0.990 | -0.024 | 0.997 |
| FC2 | -0.132 | 0.981 | 0.113 | 0.988 | -0.005 | 0.990 | -0.016 | 0.997 |
| FT8 | -0.026 | 0.981 | 0.031 | 0.988 | 0.078 | 0.990 | -0.008 | 0.997 |
| FC6 | -0.176 | 0.981 | 0.053 | 0.988 | 0.054 | 0.990 | 0.139 | 0.997 |
| F8 | 0.139 | 0.981 | 0.113 | 0.988 | -0.020 | 0.990 | -0.051 | 0.997 |
| F6 | -0.226 | 0.981 | 0.141 | 0.988 | -0.005 | 0.990 | 0.013 | 0.997 |
| F4 | 0.114 | 0.981 | 0.099 | 0.988 | -0.048 | 0.990 | 0.012 | 0.997 |
| Cz | 0.032 | 0.981 | -0.051 | 0.988 | -0.091 | 0.990 | -0.114 | 0.997 |

Values are Pearson’s correlation coefficients. PD: Panic disorder, RS: Resting state, MA: Mental arithmetic, TGC: Theta-Gamma Coupling, BDZ, Benzodiazepine, FDR: False Discovery Rate. ^a^ Data presented as oral lorazepam-equivalent dose for benzodiazepines. Benzodiazepine doses were calculated and transformed into lorazepam equivalents. Lorazepam 1mg equivalent doses: diazepam 5 mg, clonazepam 0.375 mg, alprazolam 0.5 mg, etizolam 1 mg.

**Supplementary Table 7.** The comparisons of TGC between PD patients with mild and moderate depression

| Lead | | Mild depression with PD (HAMD score 0–17) | | Moderate depression with PD (HAMD score 18–30) | | t | *p-*_FDR_ |
| --- | --- | --- | --- | --- | --- | --- | --- |
|  |  | Mean | SD | Mean | SD |  |  |
| TGC during the resting-state | AF4 | 1.46E-05 | 6.04E-06 | 1.23E-05 | 6.31E-06 | 1.069 | 0.596 |
|  | F2 | 1.46E-05 | 5.19E-06 | 1.20E-05 | 5.89E-06 | 1.377 | 0.547 |
|  | FCz | 2.79E-05 | 3.02E-05 | 1.32E-05 | 6.96E-06 | 1.900 | 0.547 |
|  | Fp2 | 1.79E-05 | 1.44E-05 | 1.14E-05 | 4.81E-06 | 1.713 | 0.547 |
|  | Fz | 2.29E-05 | 2.70E-05 | 1.26E-05 | 6.59E-06 | 1.492 | 0.547 |
|  | FC1 | 1.78E-05 | 1.34E-05 | 1.38E-05 | 8.50E-06 | 1.038 | 0.604 |
|  | AFz | 1.62E-05 | 9.17E-06 | 1.18E-05 | 4.34E-06 | 1.762 | 0.547 |
|  | F1 | 1.58E-05 | 5.03E-06 | 1.32E-05 | 6.56E-06 | 1.328 | 0.547 |
|  | Fp1 | 1.60E-05 | 1.33E-05 | 1.18E-05 | 5.53E-06 | 1.162 | 0.552 |
|  | AF3 | 1.57E-05 | 6.20E-06 | 1.23E-05 | 5.83E-06 | 1.661 | 0.547 |
|  | F3 | 2.06E-05 | 2.01E-05 | 1.23E-05 | 5.17E-06 | 1.600 | 0.547 |
|  | F5 | 1.55E-05 | 7.33E-06 | 1.20E-05 | 5.37E-06 | 1.544 | 0.547 |
|  | FC5 | 1.51E-05 | 5.74E-06 | 1.28E-05 | 5.49E-06 | 1.218 | 0.552 |
|  | FC3 | 1.35E-05 | 3.79E-06 | 1.15E-05 | 5.43E-06 | 1.297 | 0.547 |
|  | C1 | 1.60E-05 | 6.83E-06 | 1.14E-05 | 3.74E-06 | 2.434 | 0.547 |
|  | F7 | 2.03E-05 | 2.05E-05 | 1.11E-05 | 4.00E-06 | 1.758 | 0.547 |
|  | FT7 | 1.41E-05 | 6.18E-06 | 1.14E-05 | 3.97E-06 | 1.457 | 0.547 |
|  | C3 | 1.44E-05 | 5.09E-06 | 1.17E-05 | 4.28E-06 | 1.683 | 0.547 |
|  | CP1 | 1.61E-05 | 5.04E-06 | 1.18E-05 | 5.44E-06 | 2.408 | 0.547 |
|  | C5 | 1.37E-05 | 5.66E-06 | 1.19E-05 | 5.66E-06 | 0.947 | 0.625 |
|  | T3 | 2.08E-05 | 2.23E-05 | 1.09E-05 | 4.70E-06 | 1.735 | 0.547 |
|  | TP7 | 1.30E-05 | 5.45E-06 | 1.19E-05 | 5.49E-06 | 0.571 | 0.776 |
|  | CP5 | 1.30E-05 | 5.38E-06 | 1.39E-05 | 5.84E-06 | -0.475 | 0.827 |
|  | P5 | 1.32E-05 | 5.60E-06 | 1.37E-05 | 7.04E-06 | -0.238 | 0.877 |
|  | P3 | 1.49E-05 | 4.65E-06 | 1.25E-05 | 5.02E-06 | 1.440 | 0.547 |
|  | A1 | 1.22E-05 | 6.78E-06 | 1.33E-05 | 5.25E-06 | -0.541 | 0.785 |
|  | T5 | 1.18E-05 | 4.95E-06 | 1.39E-05 | 5.19E-06 | -1.174 | 0.552 |
|  | P1 | 1.40E-05 | 5.34E-06 | 1.37E-05 | 6.35E-06 | 0.130 | 0.947 |
|  | P9 | 1.26E-05 | 4.57E-06 | 1.31E-05 | 6.00E-06 | -0.297 | 0.877 |
|  | PO3 | 1.61E-05 | 1.58E-05 | 1.20E-05 | 5.16E-06 | 0.983 | 0.625 |
|  | Pz | 1.91E-05 | 2.32E-05 | 1.32E-05 | 7.81E-06 | 0.966 | 0.625 |
|  | O1 | 1.18E-05 | 4.95E-06 | 1.49E-05 | 8.40E-06 | -1.343 | 0.547 |
|  | POz | 1.65E-05 | 1.85E-05 | 1.34E-05 | 5.61E-06 | 0.636 | 0.755 |
|  | Oz | 1.25E-05 | 4.61E-06 | 1.38E-05 | 6.16E-06 | -0.674 | 0.739 |
|  | PO4 | 2.22E-05 | 3.34E-05 | 1.21E-05 | 6.86E-06 | 1.179 | 0.552 |
|  | O2 | 1.76E-05 | 2.63E-05 | 1.25E-05 | 6.78E-06 | 0.753 | 0.728 |
|  | P2 | 1.83E-05 | 2.87E-05 | 1.30E-05 | 6.14E-06 | 0.725 | 0.730 |
|  | CP2 | 1.65E-05 | 1.61E-05 | 1.39E-05 | 7.07E-06 | 0.602 | 0.767 |
|  | P4 | 1.31E-05 | 5.03E-06 | 1.45E-05 | 6.20E-06 | -0.748 | 0.728 |
|  | P10 | 1.32E-05 | 6.40E-06 | 1.09E-05 | 2.92E-06 | 1.339 | 0.547 |
|  | T6 | 1.30E-05 | 5.01E-06 | 1.25E-05 | 3.32E-06 | 0.287 | 0.877 |
|  | P6 | 1.38E-05 | 6.74E-06 | 1.36E-05 | 6.08E-06 | 0.080 | 0.971 |
|  | CP6 | 1.49E-05 | 5.21E-06 | 1.34E-05 | 6.43E-06 | 0.749 | 0.728 |
|  | A2 | 1.18E-05 | 5.04E-06 | 1.22E-05 | 5.37E-06 | -0.235 | 0.877 |
|  | TP8 | 1.47E-05 | 5.67E-06 | 1.41E-05 | 6.44E-06 | 0.302 | 0.877 |
|  | C6 | 1.51E-05 | 8.27E-06 | 1.51E-05 | 8.19E-06 | -0.012 | 0.990 |
|  | C4 | 1.48E-05 | 5.62E-06 | 1.43E-05 | 7.12E-06 | 0.244 | 0.877 |
|  | C2 | 1.44E-05 | 6.51E-06 | 1.52E-05 | 6.32E-06 | -0.382 | 0.873 |
|  | T4 | 2.02E-05 | 2.43E-05 | 1.52E-05 | 1.74E-05 | 0.681 | 0.739 |
|  | FC4 | 1.43E-05 | 4.37E-06 | 1.37E-05 | 7.45E-06 | 0.300 | 0.877 |
|  | FC2 | 1.96E-05 | 1.97E-05 | 1.38E-05 | 5.38E-06 | 1.143 | 0.552 |
|  | FT8 | 1.21E-05 | 4.29E-06 | 1.28E-05 | 5.55E-06 | -0.408 | 0.869 |
|  | FC6 | 1.35E-05 | 5.99E-06 | 1.35E-05 | 6.02E-06 | -0.015 | 0.990 |
|  | F8 | 1.83E-05 | 1.87E-05 | 1.20E-05 | 5.84E-06 | 1.296 | 0.547 |
|  | F6 | 1.51E-05 | 5.70E-06 | 1.33E-05 | 6.42E-06 | 0.871 | 0.674 |
|  | F4 | 1.66E-05 | 1.46E-05 | 1.16E-05 | 5.62E-06 | 1.276 | 0.547 |
|  | Cz | 2.67E-05 | 3.78E-05 | 1.30E-05 | 5.24E-06 | 1.440 | 0.547 |
| TGC during the MA | AF4 | 1.44E-05 | 8.71E-06 | 1.09E-05 | 5.14E-06 | 1.360 | 0.519 |
|  | F2 | 1.34E-05 | 6.51E-06 | 1.08E-05 | 4.42E-06 | 1.296 | 0.519 |
|  | FCz | 1.46E-05 | 6.65E-06 | 1.39E-05 | 5.26E-06 | 0.345 | 0.835 |
|  | Fp2 | 1.21E-05 | 5.45E-06 | 9.42E-06 | 3.68E-06 | 1.603 | 0.519 |
|  | Fz | 1.29E-05 | 5.55E-06 | 1.13E-05 | 3.35E-06 | 0.960 | 0.558 |
|  | FC1 | 1.37E-05 | 7.01E-06 | 1.25E-05 | 4.66E-06 | 0.545 | 0.782 |
|  | AFz | 1.36E-05 | 1.05E-05 | 1.03E-05 | 3.67E-06 | 1.158 | 0.539 |
|  | F1 | 1.46E-05 | 7.08E-06 | 1.25E-05 | 5.07E-06 | 0.986 | 0.558 |
|  | Fp1 | 1.16E-05 | 5.25E-06 | 9.34E-06 | 2.96E-06 | 1.496 | 0.519 |
|  | AF3 | 1.15E-05 | 5.96E-06 | 1.18E-05 | 5.12E-06 | -0.109 | 0.940 |
|  | F3 | 1.25E-05 | 6.18E-06 | 9.80E-06 | 4.51E-06 | 1.405 | 0.519 |
|  | F5 | 1.14E-05 | 3.95E-06 | 1.04E-05 | 3.73E-06 | 0.712 | 0.704 |
|  | FC5 | 1.22E-05 | 6.14E-06 | 1.14E-05 | 3.53E-06 | 0.438 | 0.808 |
|  | FC3 | 1.32E-05 | 7.48E-06 | 1.15E-05 | 4.03E-06 | 0.796 | 0.648 |
|  | C1 | 1.38E-05 | 6.47E-06 | 1.10E-05 | 3.90E-06 | 1.458 | 0.519 |
|  | F7 | 1.35E-05 | 4.41E-06 | 1.12E-05 | 4.39E-06 | 1.495 | 0.519 |
|  | FT7 | 1.22E-05 | 5.51E-06 | 1.12E-05 | 4.24E-06 | 0.563 | 0.782 |
|  | C3 | 1.38E-05 | 6.63E-06 | 1.22E-05 | 4.34E-06 | 0.815 | 0.648 |
|  | CP1 | 1.30E-05 | 5.22E-06 | 1.28E-05 | 4.36E-06 | 0.132 | 0.940 |
|  | C5 | 1.37E-05 | 6.96E-06 | 1.16E-05 | 3.46E-06 | 1.057 | 0.558 |
|  | T3 | 1.18E-05 | 4.82E-06 | 1.17E-05 | 4.53E-06 | 0.072 | 0.943 |
|  | TP7 | 1.47E-05 | 7.25E-06 | 1.10E-05 | 4.60E-06 | 1.705 | 0.519 |
|  | CP5 | 1.81E-05 | 1.29E-05 | 1.21E-05 | 4.17E-06 | 1.714 | 0.519 |
|  | P5 | 1.43E-05 | 6.94E-06 | 1.25E-05 | 3.03E-06 | 0.944 | 0.558 |
|  | P3 | 1.23E-05 | 7.26E-06 | 1.16E-05 | 5.00E-06 | 0.276 | 0.862 |
|  | A1 | 1.24E-05 | 8.70E-06 | 1.14E-05 | 4.58E-06 | 0.406 | 0.808 |
|  | T5 | 1.30E-05 | 6.93E-06 | 1.27E-05 | 3.73E-06 | 0.183 | 0.921 |
|  | P1 | 1.09E-05 | 4.28E-06 | 1.30E-05 | 3.32E-06 | -1.550 | 0.519 |
|  | P9 | 1.51E-05 | 6.76E-06 | 1.26E-05 | 4.05E-06 | 1.258 | 0.519 |
|  | PO3 | 1.35E-05 | 6.15E-06 | 1.42E-05 | 7.84E-06 | -0.274 | 0.862 |
|  | Pz | 1.53E-05 | 6.26E-06 | 1.28E-05 | 5.75E-06 | 1.185 | 0.537 |
|  | O1 | 1.51E-05 | 5.93E-06 | 1.17E-05 | 4.73E-06 | 1.800 | 0.519 |
|  | POz | 1.47E-05 | 6.00E-06 | 1.24E-05 | 6.52E-06 | 1.047 | 0.558 |
|  | Oz | 1.70E-05 | 1.19E-05 | 1.19E-05 | 4.14E-06 | 1.576 | 0.519 |
|  | PO4 | 1.51E-05 | 9.35E-06 | 1.05E-05 | 3.76E-06 | 1.774 | 0.519 |
|  | O2 | 1.59E-05 | 1.25E-05 | 1.19E-05 | 7.00E-06 | 1.075 | 0.558 |
|  | P2 | 1.17E-05 | 5.58E-06 | 1.15E-05 | 5.78E-06 | 0.097 | 0.940 |
|  | CP2 | 1.39E-05 | 7.67E-06 | 1.16E-05 | 3.83E-06 | 1.041 | 0.558 |
|  | P4 | 1.56E-05 | 9.62E-06 | 1.38E-05 | 1.07E-05 | 0.500 | 0.786 |
|  | P10 | 1.67E-05 | 1.58E-05 | 1.07E-05 | 5.95E-06 | 1.404 | 0.519 |
|  | T6 | 1.64E-05 | 1.39E-05 | 9.84E-06 | 3.47E-06 | 1.785 | 0.519 |
|  | P6 | 1.57E-05 | 1.20E-05 | 1.23E-05 | 6.26E-06 | 1.012 | 0.558 |
|  | CP6 | 1.72E-05 | 1.16E-05 | 1.30E-05 | 6.89E-06 | 1.242 | 0.519 |
|  | A2 | 1.15E-05 | 4.31E-06 | 1.29E-05 | 1.45E-05 | -0.396 | 0.808 |
|  | TP8 | 1.64E-05 | 1.41E-05 | 1.17E-05 | 4.58E-06 | 1.231 | 0.519 |
|  | C6 | 1.52E-05 | 8.31E-06 | 1.22E-05 | 3.16E-06 | 1.312 | 0.519 |
|  | C4 | 1.61E-05 | 8.98E-06 | 1.49E-05 | 8.33E-06 | 0.415 | 0.808 |
|  | C2 | 1.49E-05 | 7.54E-06 | 1.34E-05 | 5.25E-06 | 0.656 | 0.718 |
|  | T4 | 1.29E-05 | 5.57E-06 | 1.74E-05 | 1.85E-05 | -0.983 | 0.558 |
|  | FC4 | 1.47E-05 | 8.20E-06 | 1.35E-05 | 4.00E-06 | 0.511 | 0.786 |
|  | FC2 | 1.76E-05 | 8.60E-06 | 1.22E-05 | 4.27E-06 | 2.228 | 0.519 |
|  | FT8 | 1.57E-05 | 8.18E-06 | 1.20E-05 | 5.32E-06 | 1.490 | 0.519 |
|  | FC6 | 1.48E-05 | 6.52E-06 | 1.18E-05 | 3.82E-06 | 1.556 | 0.519 |
|  | F8 | 1.38E-05 | 6.45E-06 | 1.01E-05 | 3.21E-06 | 2.006 | 0.519 |
|  | F6 | 1.45E-05 | 6.89E-06 | 1.08E-05 | 4.59E-06 | 1.766 | 0.519 |
|  | F4 | 1.49E-05 | 9.76E-06 | 1.12E-05 | 4.26E-06 | 1.346 | 0.519 |
|  | Cz | 1.52E-05 | 6.81E-06 | 1.88E-05 | 2.10E-05 | -0.690 | 0.706 |

TGC: Theta-Gamma Coupling, PD: Panic disorder, HAMD: Hamilton Rating Scale for Depression, SD: Standard deviation, FDR: False Discovery Rate, MI: Modulation index, MA: Mental arithmetic.
